# An RNA-seq Based Machine Learning Approach Identifies Latent Tuberculosis Patients With an Active Tuberculosis Profile

**DOI:** 10.3389/fimmu.2020.01470

**Published:** 2020-07-14

**Authors:** Olivia Estévez, Luis Anibarro, Elina Garet, Ángeles Pallares, Laura Barcia, Laura Calviño, Cremildo Maueia, Tufária Mussá, Florentino Fdez-Riverola, Daniel Glez-Peña, Miguel Reboiro-Jato, Hugo López-Fernández, Nuno A. Fonseca, Rajko Reljic, África González-Fernández

**Affiliations:** ^1^CINBIO, Universidade de Vigo, Immunology Group, Campus Universitario Lagoas-Marcosende, Vigo, Spain; ^2^Galicia Sur Health Research Institute (IIS Galicia Sur), SERGAS-UVIGO, Vigo, Spain; ^3^Tuberculosis Unit, Department of Infectious Diseases and Internal Medicine, University Hospital Complex of Pontevedra, Pontevedra, Spain; ^4^Grupo de Estudio de Infecciones por Micobacterias (GEIM), Spanish Society of Infectious Diseases (SEIMC), Madrid, Spain; ^5^Department of Microbiology, University Hospital Complex of Pontevedra, Pontevedra, Spain; ^6^Departamento de Plataformas Tecnológicas, Instituto Nacional de Saúde, Ministério da Saúde, Maputo, Mozambique; ^7^Department of Microbiology, Faculty of Medicine, Eduardo Mondlane University, Maputo, Mozambique; ^8^ESEI - Escuela Superior de Ingeniería Informática, Edificio Politécnico, Universitario As Lagoas s/n, Universidad de Vigo, Ourense, Spain; ^9^European Bioinformatics Institute, Cambridge, United Kingdom; ^10^CIBIO/InBIO - Research Center in Biodiversity and Genetic Resources, Universidade do Porto, Vairão, Portugal; ^11^St. George's, University of London, London, United Kingdom

**Keywords:** tuberculosis, latent tuberculosis, RNA-seq, machine-learning, TB progression

## Abstract

A better understanding of the response against Tuberculosis (TB) infection is required to accurately identify the individuals with an active or a latent TB infection (LTBI) and also those LTBI patients at higher risk of developing active TB. In this work, we have used the information obtained from studying the gene expression profile of active TB patients and their infected –LTBI- or uninfected –NoTBI- contacts, recruited in Spain and Mozambique, to build a class-prediction model that identifies individuals with a TB infection profile. Following this approach, we have identified several genes and metabolic pathways that provide important information of the immune mechanisms triggered against TB infection. As a novelty of our work, a combination of this class-prediction model and the direct measurement of different immunological parameters, was used to identify a subset of LTBI contacts (called *TB-like)* whose transcriptional and immunological profiles are suggestive of infection with a higher probability of developing active TB. Validation of this novel approach to identifying LTBI individuals with the highest risk of active TB disease merits further longitudinal studies on larger cohorts in TB endemic areas.

## Introduction

Tuberculosis (TB), the infectious disease caused by *Mycobacterium tuberculosis* (*Mtb*), is the leading cause of death from a single infectious agent worldwide (1). Despite being a long known disease, the approaches for TB diagnosis and therapy available to date have not yet been able to successfully control this world health problem. It has been estimated that 1.7 billion people are latently infected (LTBI) with *Mtb*, from whom a small percentage will develop active TB disease during their lifetime.

Although the classification of the *Mtb* infection status is currently dichotomic, divided into latent or active TB, it is clear that there is a spectrum of different TB infection stages (2, 3). The spectrum includes, among others, people who have cleared the infection, latently infected individuals, or those with a subclinical or incipient TB infection. Unfortunately, the Tuberculin Skin Test (TST) and Interferon Gamma Release Assays (IGRA) cannot differentiate between LTBI and active TB, nor identify the different stages of *Mtb* infection, or the people at higher risk of developing active disease. Furthermore, the diagnosis of LTBI using these tests can lead to both false positive and negative results (4). Although IGRA provides a greater specificity over TST (5), T-cell responses to mycobacterial antigens persist even after the infection has been cleared. As a result, the LTBI diagnosis may include a broad spectrum of individuals, from those that have cleared the infection to those with a high risk of progression to active TB.

The screening of *Mtb*-infected individuals is of great importance for TB prevention programs. In order to control and eliminate TB disease worldwide, the World Health Organization's (WHO) “End Tuberculosis Strategy” recommends the early diagnosis and treatment of LTBI people at higher risk of infection (6). However, the treatment regimens for latent TB infection are not devoid of potential toxicity and drug-related adverse effects. Since the estimation is that only 5–10% of LTBI patients will eventually progress to active TB disease, it is desirable to improve the identification of those individuals with higher risk of TB progression, as they would benefit the most from receiving anti-TB treatment.

The gene expression profiling has proved to be a potent tool for the identification of different events involved in TB infection. Several studies have been conducted using whole-genome microarrays (7–12) and less frequently RNA-seq (13) that proves the suitability of transcriptomics to identify the key mechanisms of TB infection. However, the identification of the events that precede the progression to active TB are not yet fully understood. Although recent works have provided information of these mechanisms (14, 15), further studies are needed to identify common features within cohorts from different locations. In addition, new approaches that allow the identification of different profiles within LTBI individuals without the requirement of a long-lasting follow-up studies are also of interest.

In this work, we have conducted an RNA-seq gene expression study in patients recruited in two different countries (Spain and Mozambique) in order to identify a robust signature of the mechanisms that define the infection. The gene expression profile was used to study the heterogeneity within LTBI individuals applying a machine-learning based procedure. We found a percentage of these individuals showing immunological and transcriptomic features of active TB profile that suggest a correlation with the events that take place before progressing to active TB. Based on our results, we propose that there is a specific list of genes expressed in peripheral blood that could discriminate between the two groups of LTBI persons (*NoTB-like* and *TB-like*). The early identification of individuals with a *TB-like* profile, with higher probability for progressing to TB, opens the possibility to target more accurately the recommendation for receiving preventive TB treatment.

## Materials and Methods

### Recruitment of Study Participants

The RNA-seq analysis was performed on samples from two newly recruited cohorts, one from Galicia (Spain) and the second from a high-burden TB country (Mozambique), used for validation purposes. Both cohorts included pulmonary TB patients and their contacts, classified as uninfected (NoTBI) and LTBI contacts.

Participants were recruited between September 2015 and February 2018 at the Tuberculosis Unit in the “Complexo Hospitalario Universitario de Pontevedra” (Galicia, Spain) and the “Centro de Saúde da Machava II” and the “Centro de Saúde de Mavalane,” both based in Maputo, Mozambique.

Contacts were diagnosed either as LTBI or uninfected (NoTBI) according to the Spanish consensus for TB diagnosis (16) based on the results of the TST and/or the IGRA QuantiFERON®-TB Gold in-tube (QFT-GIT) test. In the case of the Mozambican cohort, LTBI or NoTBI diagnosis was based only on the IGRA results. In those patients with an initial negative result, this was repeated 8–10 weeks after the last possible exposure to *Mtb* in order to rule out a false negative result before the “window period” (17). Active TB disease was ruled out in TST/IGRA positive contacts if they showed no clinical manifestations of the disease, a normal chest X-ray and negative microbiological readout.

The study was approved by the Galician Ethics Committee (registry number: 2014/492) and the National Bioethics Committee for Health of Mozambique (reference number 298/CNBS/2015). All Participants gave their written informed consent after appropriate counseling prior to enrolment in the study.

### Inclusion and Exclusion Criteria

Newly diagnosed pulmonary TB patients with microbiologically confirmed *M. tuberculosis* in respiratory specimens were recruited prior to initiation of anti-TB treatment or within the first 5 days of treatment due to logistic reasons. TB contacts included healthy people exposed to a pulmonary microbiologically confirmed TB index case. In order to have a controlled cohort of people not suffering from any other condition that could interfere in the TB study, people matching the exclusion criteria summarized in Table 1 were not considered for study.

**Table 1:** Exclusion criteria for participants' recruitment.

**Exclusion Criteria**	
All participants	Having received anti-TB treatment before HIV co-infection irrespective of CD4 count TST (Tuberculin Test) in the last 3 months Immunosuppressive treatment (Prednisone > 10 mg/day or equivalent; TNF blockers; cancer chemotherapy). Inhaled corticosteroids (At least during the previous month). End Stage Renal Disease Diabetes Alcoholism (as confirmed by the attending physician) Patients with autoimmune disorders or any other immunosuppressive state (as confirmed by the attending physician) Pregnant women Unwilling to participate Being under 18 years old*.
Contacts only	Previous TB diagnosis Previous positive TST/IGRA documented Previous old healed lesion on chest radiography Recent (<3 months) vaccination with live-attenuated strains Any other active infection during the previous month IGRA result indeterminate

### Tuberculin Skin Test and Interferon Gamma Release Assay Test

TST or Quantiferon^TM^ TB Gold In-Tube (QFT) (Cellestis Ltd, Carnegie, Australia) were both performed at the first visit to the clinic.

TST was conducted according to the Mantoux method, with 2 units of tuberculin RT-23 (PPD, Statens Serum Institute, Copenhagen, Denmark), following the standardized protocol. The induration diameter was measured at 48–72 h. A positive TST was defined as an induration of ≥ 5 mm following Spanish national guidelines (16). TST conversion to positivity was indicated by an increase in induration diameter of at least 10 mm over a previously negative TST result.

The TB Quantiferon Gold Kit was used to detect the presence of Interferon gamma produced by T cells in response to TB antigens, following the manufacturer's instructions. Samples were previously frozen and stored at −80°C until analysis, 3–4 weeks later. The cut-off value for a positive test was 0.35 IU/mL.

### Blood RNA Isolation and Sequencing

Whole blood RNA was isolated from 2 ml of blood collected in EDTA-coated vacutainer tubes (BD Vacutainer, USA). After removing the plasma fraction, RNA was isolated using the QIAamp RNA Blood Mini kit (Qiagen; Hilden, Germany) following the manufacturer's instructions. Isolated RNA was stored at −80°C until their analysis and a small fraction was used to evaluate its quality. The RIN value was assessed using an Agilent 2100 Bioanalyzer and the Agilent RNA 600 Nano Kit (Agilent Technologies; CA, USA). Only samples with a RIN value > 7 and a minimum concentration of 20 ng/mL were sequenced.

Whole blood RNA sequencing was performed on an Ion Proton sequencer (Ion Torrent, Thermo Fisher Scientific; CA, USA). Poly(A)-mRNA fraction was enriched processing 400–500 ng of total RNA with the Dynabeads® mRNA DIRECT™ Micro Kit (Thermo Fisher Scientific; CA, USA) according to the manufacturer's protocol. The enriched mRNA was then used to prepare barcoded libraries with the Ion Total RNA-Seq Kit v2 (Life technologies- Thermo Fisher Scientific; CA, USA) following the manufacturer's instructions. Library construction and sequencing were performed by the personnel of the Genomic Service at the Scientific and Technological Research Assistance center (CACTI) (Vigo, Spain). Fastq files were then used to quantify the gene expression.

RNA-seq data generated and analyzed in this work have been deposited in the ArrayExpress database at EMBL-EBI under the accession number E-MTAB-7830.

### Gene Expression Quantification and Downstream Analysis

Single-end raw reads were quantified following the irap pipeline version 0.8.5.p8 (18), using Kallisto (19) and the reference genome GRCh38 (release 90). Differentially expressed (DE) genes between groups were identified using DESeq2 (20) R-package (version 1.18.1). The default parameters of DESeq2 were used, with the TB group as the condition following the model design: “design = ~ condition.” Genes with an adjusted *p*-value (*p.adj*) < 0.01 or < 0.05 and an absolute log2(Fold-change) >1 were considered significant in terms of differential expression. In order to rule out the influence of having started the anti-TB therapy, we compared the TB patients that were within the first 5 days of treatment, with those that had not started it. No DE genes were found between them (data not shown), hence all TB patients were studied together in following steps.

The list of DE genes was used to perform a pathway enrichment analysis using the ReactomePA R package (21) and a hierarchical clustering analysis with the pheatmap R package (version 1.0.12). Normalized counts of the DE genes were used as the input, obtained from the DESeq2 Variance stabilizing Transformation (VST) function. Rows (i.e., genes) were scaled using the pheatmap “scale = row” parameter.

### Machine Learning-Based Class-Prediction Analysis

The free software WEKA (22) was used to conduct a class prediction study based on the DE genes derived from the Spanish cohort (train set). The train set was used to evaluate the performance of three candidate algorithms (Naïve Bayes, Random Forest and SMO) using a Leave-one-out cross-validation (LOOCV) procedure. In order to avoid gene selection biases, each round of the LOOCV included: (*i*) a new DE analysis using DEseq2 over the train samples (n-1) (*ii*) identification of the DE genes derived from the train samples; (*iii*) the model training using the train samples and the selected DE genes and (*iv*) the evaluation of the model using the remaining test sample. The algorithm with the best performance in the LOOCV was selected and a classification model was built using the expression levels of the DE genes derived from the analysis of the train set. The model was validated on the test set (Mozambique). The validated model was used to further classify the LTBI samples based on the expression of selected genes that differentiate between (confirmed) infected and uninfected people.

## Analysis of Circulating Leukocytes and Protein Concentrations in Blood

The different distribution of circulating leukocytes and selected protein concentrations in blood from LTBI participants were further analyzed.

The leukocyte count was performed using a starting volume of 165 μL of whole blood on a hematology analyser (Beckman Coulter DXA1 800; CA, USA) following the manufacturer's instructions. The absolute number of white blood cells was expressed on millions of cells per mL or in percentage of total population [(number of cells from a specific population / total number of cells) ×100].

Serum samples were obtained from 10 mL peripheral venous blood collected in serum separator tubes SST II Advance (Vacutainer, BD; Plymouth, UK) and stored at −80°C until its use. The following protein concentration was evaluated using customized Milliplex kits (Merk, Millipore; USA) following the manufacturer's instructions: IL-6, IL-7, IP-10, TGFα, TNFα, BCA-1, and IL-27. Data from the reactions were acquired with a MagPix device (Luminex; Austin, Texas, USA) with the xPonent 4.2 software. A calibration curve was built with this software based on the standards' concentrations and median fluorescence intensity and used to obtain the concentration of each sample (pg/ml). Leukocyte counts and multiplex data were analyzed using the non-parametric Mann-Whitney test. Differences were considered significant when the *p* value was <0.05. Statistical analysis was performed in PRISM (GraphPad Software v6, San Diego, California).

## Results

A total of 96 individuals in the Spanish cohort and 62 in Mozambique were recruited during this period (Table 2).

**Table 2:** Demographical composition of the Spanish and Mozambican cohorts.

	**NoTBI**	**LTBI**	**TB**
**Spain**			
Total	41	27	28
Males (%)	19 (46.3)	16 (59.3)	23 (82.1)
Age mean (range)	39 (19–76)	48 (19–71)	41 (21–72)
**Mozambique**			
Total	9	16	37
Males (%)	4 (44.4)	7 (43.8)	25 (67.6)
Age mean (range)	35 (9–80)	32 (8–59)	32 (13–61)

### Different Gene Expression Profile Between Active TB Patients and Their Contacts

A preliminary evaluation of the gene expression profile performed by a principal component analysis (PCA) showed that active TB patients presented marked differences compared to both their contact groups (LTBI and NoTBI) in the two settings. However, the expression pattern across contacts did not show a clear separation between the two subgroups (Figure S1). No gender bias was observed in the samples distribution along the PCA (Figures S1C,D).

### Differential Expression Analysis

**Figure 1:**
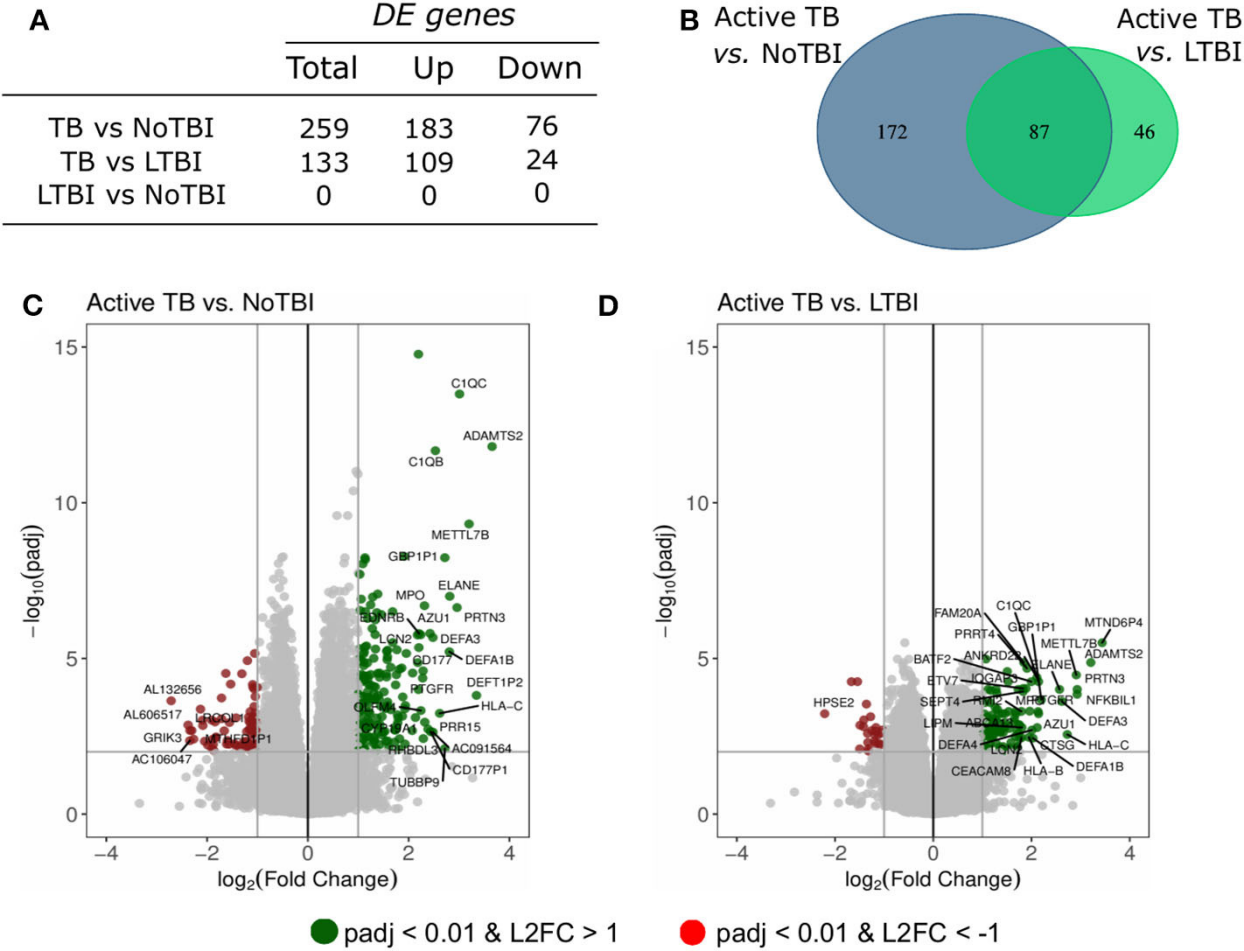
Summary of the differentially expressed (DE) genes in the pairwise contrasts comparing the TB study groups. **(A)** Total number of DE genes (adjusted *p* < 0.01 and absolute Log2 Fold Change (L2FC) >1) and number of up- or down-regulated genes. **(B)** Venn diagram with the overlapping genes between the different signatures. The volcano plots highlight the genes with significant differences between **(C)** active TB patients vs. uninfected (NoTBI) contacts and **(D)** active TB patients vs. contacts with latent infection (LTBI). The Top-30 most modulated (genes with the greatest absolute fold change) genes were labeled.

A differential expression analysis was performed using the data from the Spanish volunteers (training set). We performed pairwise comparisons between the three groups and found 259 DE genes between Active TB and NoTBI contacts (Figure 1C and Table S1) and 133 DE genes between Active TB patients and LTBI contacts (Figure 1D and Table S2). As shown in the Venn diagram (Figure 1B), these two signatures have 87 genes in common. When we compared the two contact groups between them, we could not find any gene with significant differential expression.

### Biological Processes Involved in TB Infection

**Figure 2:**
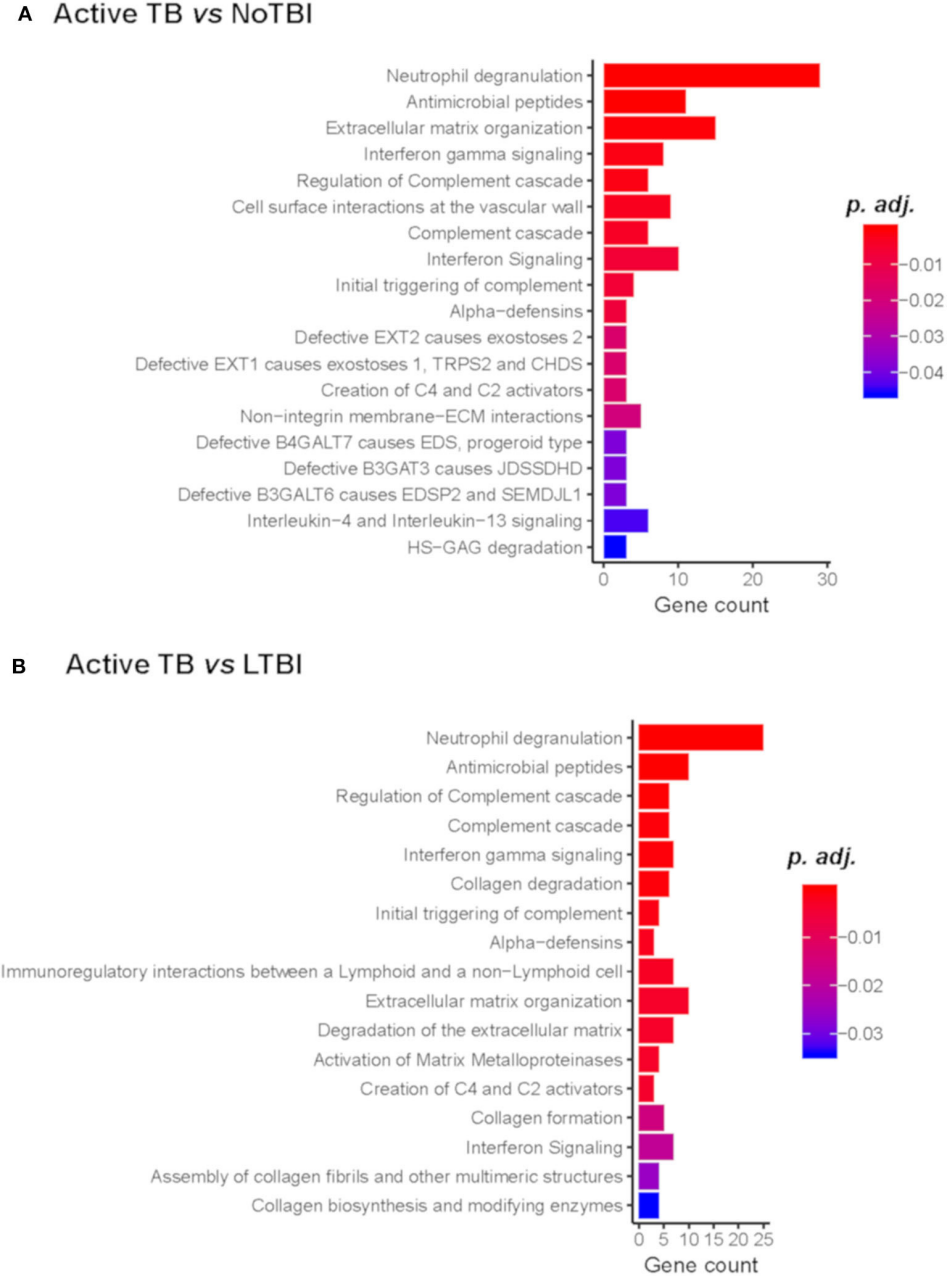
Reactome Pathway Enrichment Analysis of genes differentiating Active TB vs. NoTBI **(A)** and vs. LTBI **(B)**. The Gene count indicates the number of genes from the input list found on each pathway. The adjusted p-value (p.adj) indicates the significance of the enrichment.

A pathway enrichment analysis showed that common pathways differentiate active TB from NoTBI and LTBI contacts (Figure 2) (Tables S3, S4). These included the neutrophil degranulation cascade; expression of several defensins and antimicrobial peptides; the complement cascade; interferon (type I and II) signaling; or the activation of matrix metalloproteinases and degradation of collagen and extracellular matrix. The majority of the genes involved in these pathways were up-regulated in active TB patients.

Other genes up-regulated in active TB compared to either one or both contact groups included genes involved in the B-cell function (*MZB1* and *CD24*); Vitamin B12 carriers (*TCN1* and *TCN2*); T-cell regulation (*PDCD1LG2, CD274* and *VSIG4*) or cell division and migration, among others. In addition, an unexpectedly high number of genes coding for immunoglobulin chains were up-regulated in active TB (*p.adj* < 0.01) compared to NoTBI, but not to LTBI contacts.

### Different Gene Clusters Define the Expression Profile of TB Study Groups

**Figure 3:**
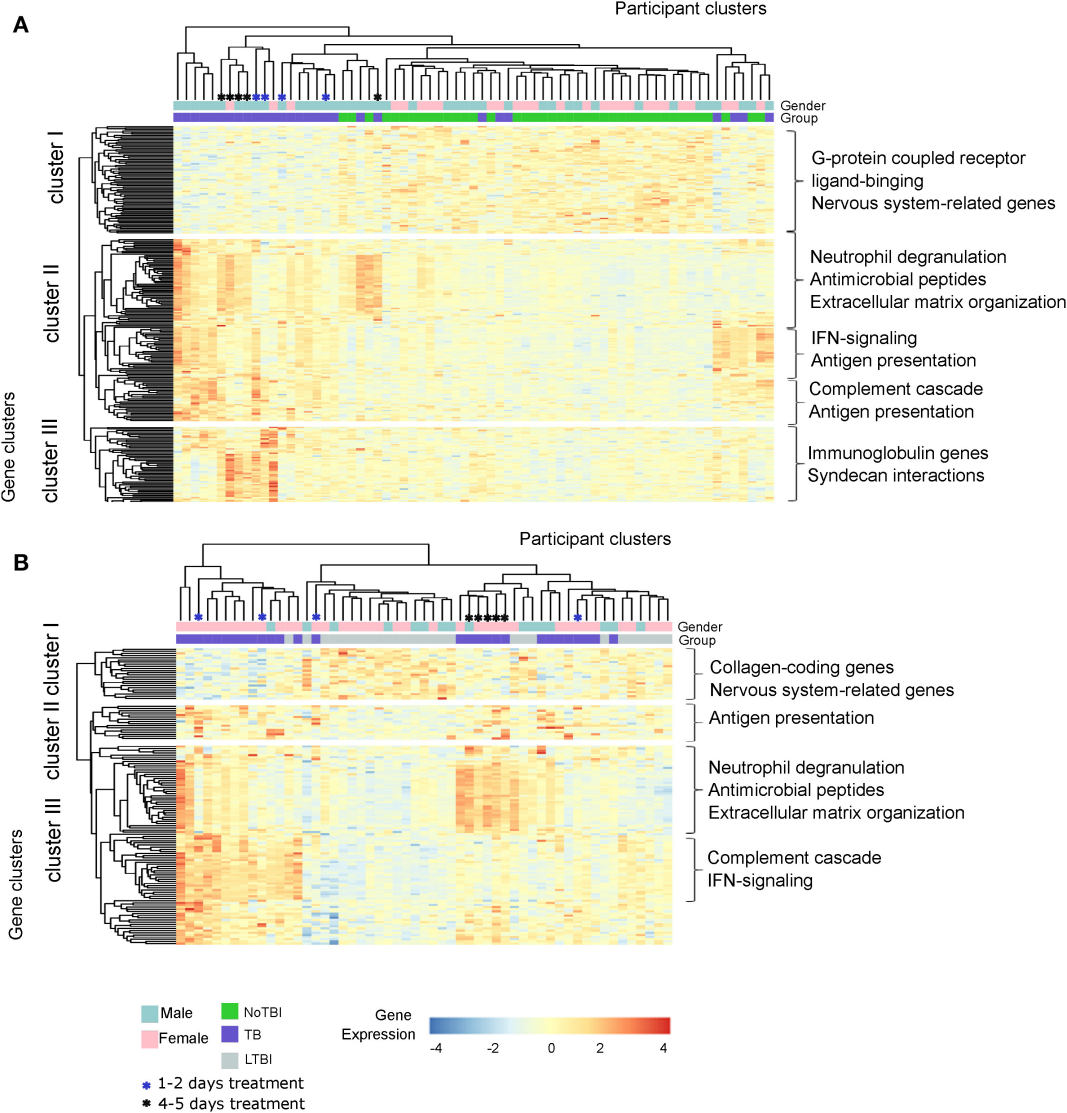
Hierarchical Clustering analysis of the active TB patients and their contacts from the Spanish cohort based on the expression of the differentially expressed genes. **(A)** Heatmap based on the 259 DE genes between Active TB and NoTBI contacts. **(B)** Heatmap based on the 133 genes between active TB and LTBI contacts. Each column of the heatmaps represents one sample and each row represents one gene. Both the samples and the genes have been clustered based on the similarity of their expression pattern. The color of the cells indicates the expression of each gene for the corresponding sample. The main enriched pathways related to each gene cluster are included.

A hierarchical clustering analysis demonstrated that active TB patients and NoTBI contacts could be differentiated based on the expression pattern of the 259 DE genes signature, with just a few exceptions (Figure 3A). On the other hand, active TB and LTBI formed three separated groups based on the expression of the 133 DE genes (Figure 3B). These clustering patterns were verified in the setting from Mozambique (Test Set, Figure S2). It should also be noticed that TB patients that were under treatment for 4–5 days before inclusion tend to cluster together (Figures 3A,B).

This analysis also showed different gene clusters within groups. Part of the active TB patients were characterized by up-regulation of genes involved in neutrophil degranulation, antimicrobial peptides and the extracellular matrix organization (Figure 3A cluster II and Figure 3B cluster III); other patients had a profile with a higher expression of genes of the interferon (IFN)-signaling, antigen presentation or the complement cascade (Figure 3A cluster II and Figure 3B cluster III) while others overexpressed genes from all these events. The differences between active TB and NoTBI contacts also included an independent cluster of immunoglobulin chain-coding genes (Figure 3A cluster III).

### Heterogeneity of Transcriptional Profiles Within the LTBI Group

Our results suggested a heterogeneous transcriptional profile within LTBI patients. On the one hand, the lack of DE genes when compared to NoTBI contacts suggests a greater proportion of people with similar profile to uninfected contacts. On the other hand, a proportion of LTBI contacts clustered together with active TB patients (Figure 3B), indicating similarity between them. Altogether, this suggests two different profiles within LTBI contacts.

**Figure 4:**
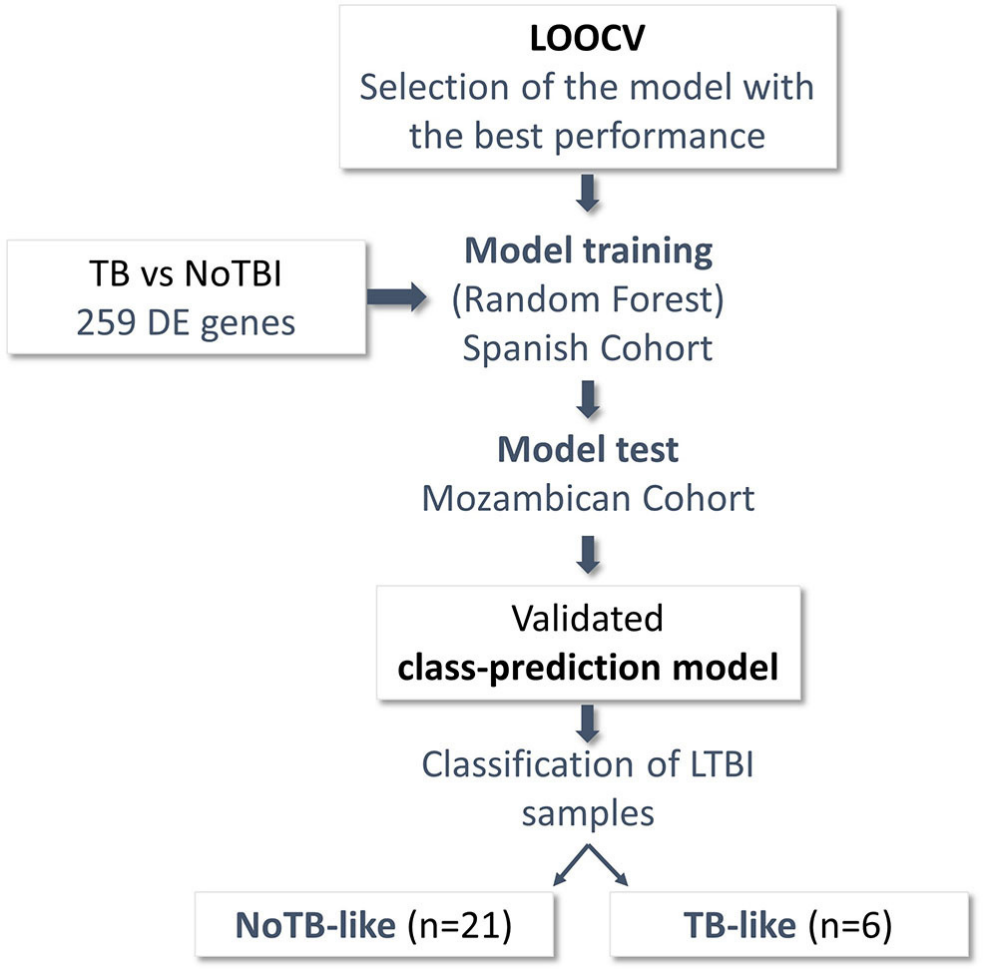
Schematic representation of the steps followed to classify the LTBI samples. Differentially Expressed (DE) genes between patients with confirmed infection (28 Active TB) and uninfected individuals (41 NoTBI) were used to create a class-prediction model. A leave-one-out Cross-validation (LOOCV) was used to select the classification algorithm. Random Forest algorithm, the one with the best performance in the LOOCV, was used to build a class-prediction model using the samples from the Spanish cohort (training set) as an input. The model was validated on an independent Mozambican cohort (test set). The validated model was then applied to the LTBI samples from the Spanish cohort to study their similarity to either the active TB patients or the uninfected contacts. Two subgroups were identified within the LTBI contacts, named *TB-like* and *NoTB-like*.

In order to study this heterogeneity and identify the LTBI contacts that could really have an infectious process, these participants were classified based on the expression of the 259 genes that differentiated active TB from NoTBI, as shown in Figure 4. For that, a Random Forest algorithm was selected based on the LOOCV result (84% accurately classified samples, 85% sensitivity, 82% specificity) to create a classification model. The model was validated in the independent cohort from Mozambique (test set), showing an accuracy of 89% correctly classified instances and 89% sensitivity (Table S5).

**Figure 5:**
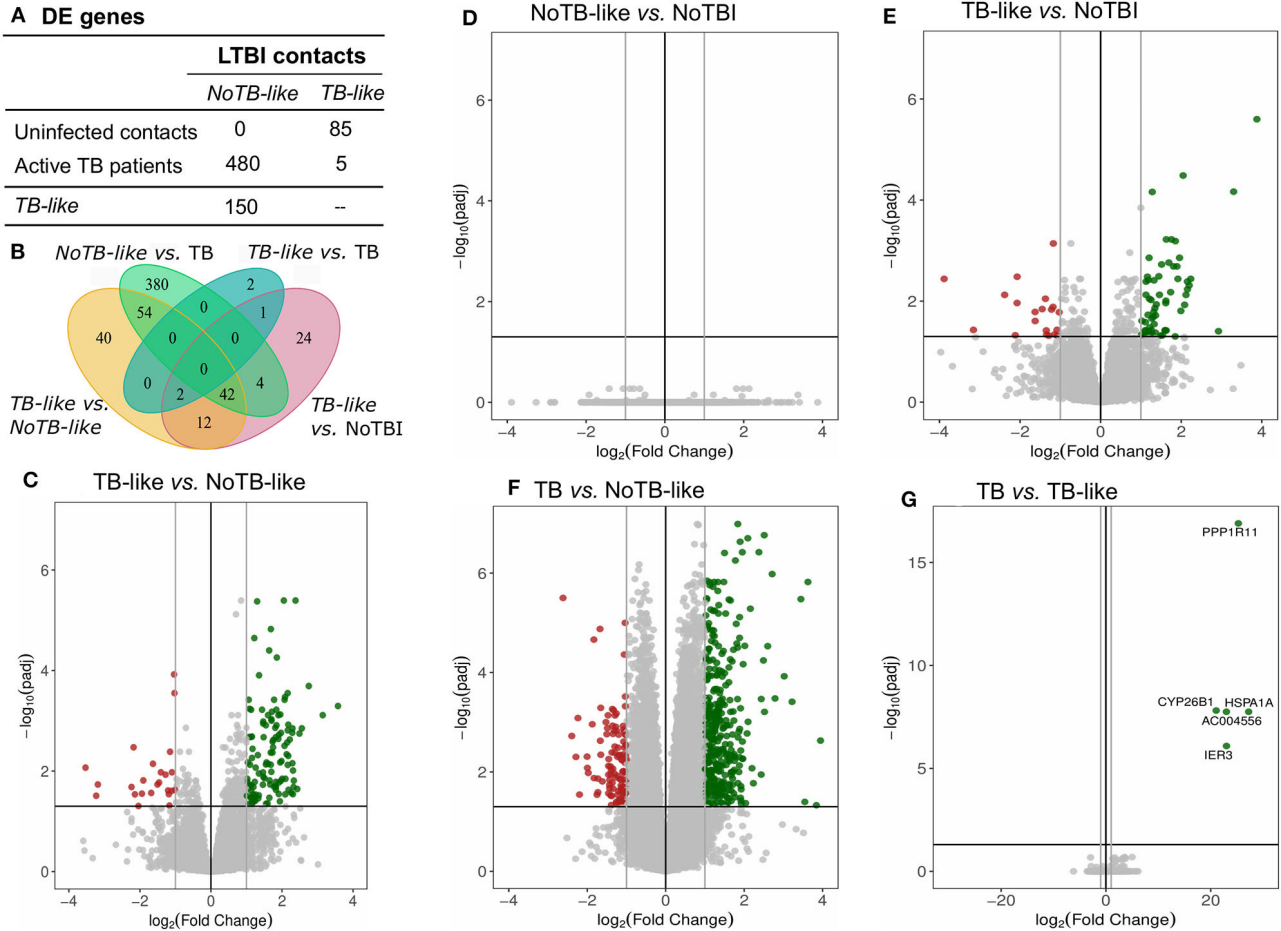
Genes differentially expressed in LTBI subgroups compared between them and to NoTBI and active TB patients. **(A)** Table summarizing the number of DE genes on each pair-wise comparison (columns vs. rows). **(B)** Venn diagram showing the overlapping genes between signatures. **(D–G)** Volcano plots highlighting the genes with significant (adjusted *p* < 0.05) fold Change between groups. Up-regulated genes (Log2(Fold Change) >1) in green and down-regulated genes (Log2(Fold Change) < -1) in red. The differential expression analysis was made using the DESeq2 R package, comparing all annotations of the reference genome (34947 annotations). Genes PPP1R11 and AC004556, which showed a log2(Fold Change) < -20, are not represented in the volcano plot **(C)**.

LTBI samples were classified applying this model, resulting in 22,2% of individuals classified as infected (*TB-like*) and the remaining 77,8% as uninfected (*NoTB-like)*. The different expression profile between the *TB-like* and the *NoTB-like* subgroups were further explored, but comparing the expression of all genes annotated on the reference genome (34947 annotations). A total of 150 DE genes (*p.adj* < 0.05) were found between these two groups (Figures 5A,C). Moreover, *NoTB-like* contacts presented no DE genes compared to NoTBI (Figures 5A,D) but 480 DE genes compared to active TB patients (Figures 5A,F). On the other hand, *TB-like* contacts presented great differences compared to NoTBI contacts (Figures 5A,E), but not compared to active TB patients (Figures 5A,G). A Venn diagram (Figure 5B) showed that there is an overlap of 96 genes between those that differentiate the *NoTB-like* group from both TB and TB-like. Likewise, there is an overlap of 56 genes between those differentiating *TB-like* and both NoTBI and NoTB-like.

**Figure 6:**
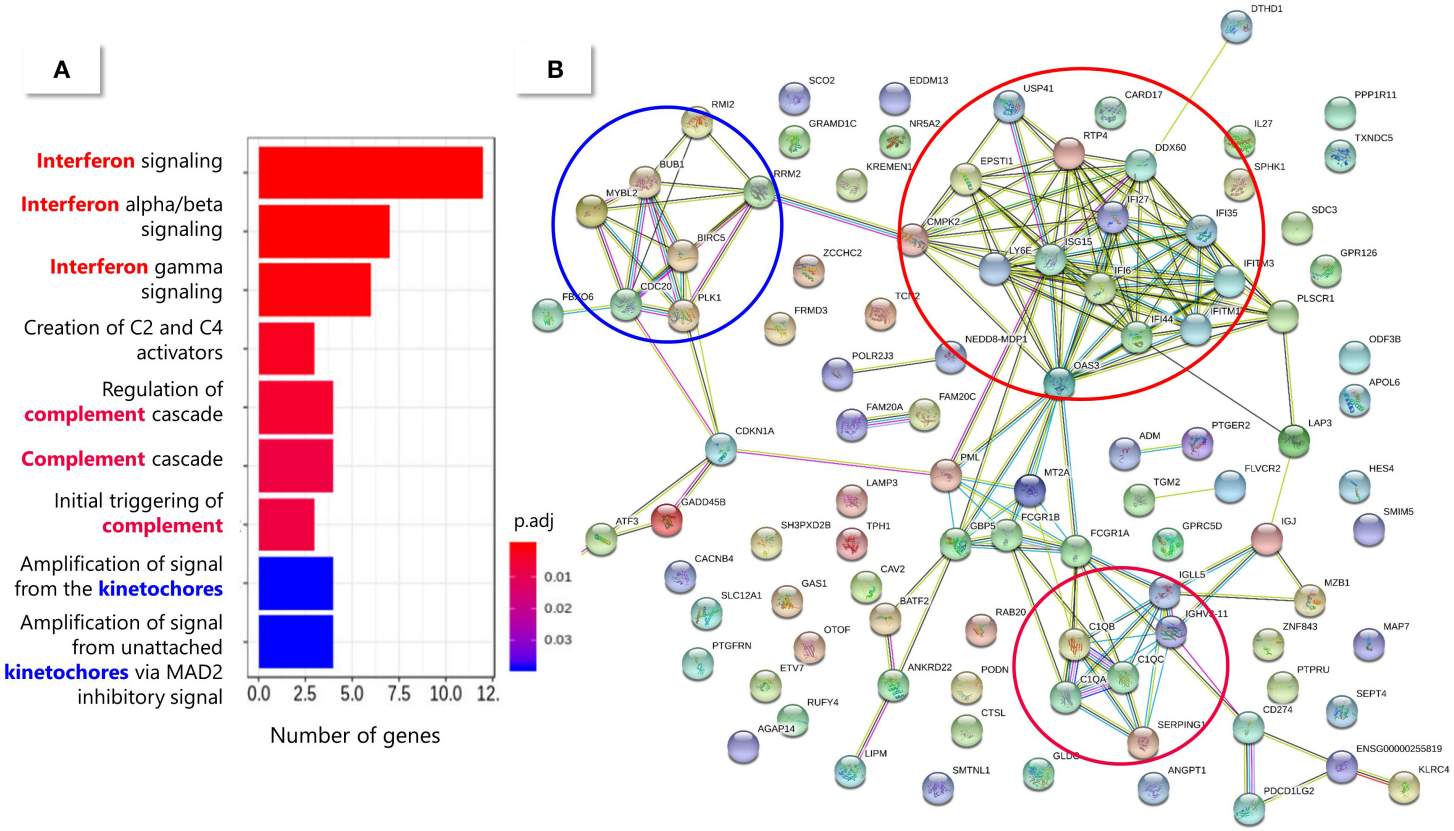
Pathway enrichment analysis of the 150 DE genes between *TB-like* and *NoTB-like* subgroups. **(A)** Enriched pathways from the Reactome database. The color of the bars indicates the significance [adjusted p-value (padj)] of the enrichment. **(B)** String protein association network. Each node (circle) represents a protein (identified by their coding gene) and the edges represent protein interactions. The main enriched pathways have been circled in a matching color with the correspondent Reactome pathway.

The 150 DE genes differentiating *TB-like* and *NoTB-like* contacts were mostly up-regulated in the *TB-like* subgroup (Figure 5C and Table S6). A pathway enrichment analysis showed that the interferon (type I and II) signaling and the complement cascade were the main processes explaining these differences, followed by the kinetochores signaling (Figure 6). Also, as in the case of TB patients, an unusually high number of genes coding for immunoglobulin chains (41 in total) were up-regulated in the *TB-like* subgroup, along with other genes related to the effector function of B cells (*MZB1, JCHAIN*) and immunoglobulin receptors (*FCGR1A* and *FCGR1B*).

This expression profile was suggestive of an infectious process in the *TB-like* group. However, according to local and WHO guidelines, treatment is indicated in LTBI contacts (16, 23), so we could not investigate their progression, or not, to active TB. In order to overcome this limitation, we used the 16-gene signature proposed by Zak et al. (14) to identify people at risk of developing active TB in our LTBI patients. An unsupervised hierarchical clustering analysis (Figure S3) showed that 5 out of the 6 *TB-like* individuals clustered together based on the expression of this 16-gene risk signature in a cluster that also includes 2 *NoTB-like* individuals. The remaining 19 individuals classified by our model as *NoTB-like* are clustered in a different group, which includes one *TB-like* individual according to this signature.

### LTBI Subgroups Present Immunological Differences

The two LTBI subgroups also showed differences in immunological parameters that were studied as part of the TB profile in our laboratory (Table 3). These data showed that the *TB-like* contacts had higher levels of leukocyte counts, higher percentage of circulating monocytes and also higher concentration of IL-6, IL-7, IP-10, TGFα and IL-27 in serum, compared to *NoTB-like* contacts (*p* < 0.05).

**Table 3:** Immunological variables in *TB-like* and *NoTB-like* patients.

**Variables**	**NoTB-like**	**TB-like**	***p***
**Cells**			
Leukocyte count (10^6^ cells/mL)	6058.57	7700	**0.029**
Neutrophils (%)	57.95	59.83	1
Lymphocytes (%)	31	26.83	0.413
Monocytes (%)	7.67	9.33	**0.043**
Eosinophils (%)	2.48	3	0.809
Basophils (%)	0.76	0.5	0.224
**Cytokines (PG/ML)**			
IL-6	42.26	93.36	**0.048**
IL-7	6.4	23.58	**0.02**
IP-10	240.12	438.99	**0.016**
TGFα	9.91	25	**0.022**
TNFα	34.84	32.51	0.143
BCA-1	23.38	24.84	0.34
IL-27	317.47	579.14	**0.013**

*Bold values indicate those that were statistically significant*.

## Discussion

The present study aimed to identify mechanisms of the immune response against TB infection that provide a better understanding of the disease and help with the identification of different profiles within the latently infected contacts.

In our work, we identified a robust 259-gene signature that differentiates active TB from uninfected contacts and a 133-gene signature to discriminate active TB and LTBI. Our results showed that one of the most important innate effector mechanisms in TB patients are the neutrophil degranulation cascade and the expression of antimicrobial peptide genes, in agreement with previous works (7, 15). Among genes coding for antimicrobial peptides we found several defensins, which are believed to play a role against *Mtb* infection (24, 25), and metalloproteinases, demonstrating their role in TB pathogenesis (26, 27). We also found genes that could be related to intracellular bacilli survival inside macrophages (*ORL1*) (28) or genes (*TCN1* and *TCN2*) coding for two carriers of cobalamin (vitamin 12), a metabolite that could play a role in *Mtb* pathogenesis (29). A greater expression of those carriers in active TB patients could benefit the mycobacteria survival inside the host by enhancing Vitamin B12 uptake. Other genes showing higher expression in TB patients were syndecans (*SDC1, SDC3*, and *SDC4*), suggesting a role for these molecules during TB infection, and the complement cascade and type I and II interferon signaling, supporting previous transcriptomic studies (8, 13, 30). Genes from the complement cascade have been seen to be substantially down-regulated during the first week of treatment (31). Related to this, we observed that the small proportion of patients under 4–5 days of treatment included in our work tended to cluster together and showed a lower expression of these genes, as seen in the heatmap. However, despite these patients, genes from the complement cascade were amongst the top-30 most up-regulated genes in the TB signature. This indicates the robustness and importance of these genes during TB infection.

Our results also showed a high expression of genes coding for immunoglobulin chains in active TB, not highlighted in previous transcriptomic analysis (7–9, 12, 32). A greater expression of these, and other genes such as *MZB1* or immunoglobulin receptors *FCGR1A*, a proposed hallmark of TB disease (8), and *FCGR1B* in active TB patients, indicate the involvement of B cells in TB infection. Active TB signature was also characterized by a higher expression of genes involved in T cell regulation (33), including the Programmed Cell Death 1 Ligand 1 (*CD274*) and 2 (*PDCD1LG2*), in agreement with Wang et al. (34).

The gene signatures derived from the Spanish cohort showed a similar clustering pattern and good classification accuracy with the Mozambique setting, indicating a robust expression profile associated with TB disease. This signature was not only used to provide a classification tool that differentiates confirmed infection from uninfected people, but also, as a novelty of our work, it provided a tool for the identification of different profiles within LTBI group by machine learning.

Latent TB Infection diagnosis is currently based on the evidence of immune memory against *Mtb*, without microbiological, radiological, or clinical evidence of active TB. The current tests, TST and IGRA, pose a very low Positive Predictive Value to predict development of active tuberculosis (35), and they do not differentiate between persistent and resolved latent infection nor do they discriminate those infected patients with higher risk for progression to TB disease (36). As a result, LTBI individuals can include people that may not have the infection anymore.

Our work showed that at least two profiles can be identified within LTBI contacts. The majority of them (77.8%) showed a transcriptional profile similar to that of uninfected contacts, and we referred to them as *NoTB-like*. The second subgroup (22.2%), on the other hand, showed a similar gene expression profile to those patients with microbiologically confirmed TB. Hence, we named them *TB-like*. Our hypothesis is that *TB-like* contacts, which present features of TB disease, would be those at higher risk of developing active TB. In this case, they would benefit the most from receiving preventive treatment.

Although the expression profile of *TB-like* contacts presents similarities with that from active TB, there were also some discrepancies. For instance, genes related to neutrophil degranulation or antimicrobial peptides were not part of their expression profile. This suggests that *TB-like* contacts may have started the activation of immune mechanisms involved in controlling the infection, but have not progressed to the later events that take place during the active killing of replicative mycobacteria. This supports the idea that *TB-like* contacts would be at the initial stages before progression to active TB.

The main limitation of our hypothesis is that progression to active TB in *TB-like* individuals could not be verified, as all LTBI patients received Isoniazid preventive treatment in accordance with local guidelines. However, several data in the literature support our findings and indicate the suitability of our approach. We showed that, with a few exceptions, the two subgroups identified here as *TB-like* and *NoTB-like* could be separated in two different clusters based on the expression of the 16-gene risk signature from Zak et al. (14). These genes, that were proposed to identify those individuals at risk of developing active TB, were up-regulated in our *TB-like* subgroup, which could suggest its correspondence with Zak's progressors. In addition, the expression profile identified in those progressors in the most proximal stage to the disease onset (15), was also in agreement with our findings. Like in our study, they identified Type I/II interferon and complement genes to be involved in early stages before progression to active TB, while expression of lymphoid, monocyte and neutrophil genes were found more proximal to the disease onset. Our results also correlate with those from Gupta et al. (37), who highlighted the importance of IFN and TNF signaling pathways amongst 40 transcripts derived from a meta-analysis of publicly available whole blood mRNA signatures proposed to identify incipient TB (who could correspond to our *TB-like* group). On the other hand, genes coding for molecules of the complement cascade, with special importance of C1q, along with Fcγ receptors (up-regulated in our *TB-like* subgroup), were also described to be up-regulated during subclinical TB, related with a greater presence of antibody/antigen complexes (38). *C1QC* was also proposed as a promising biomarker to detect TB progressors when used in combination with *TRAV27* (39). These, along with our own results, suggest the importance of the complement signaling during the early events of the disease.

The identification of common patterns with previous studies gives the notion that the machine-learning approach proposed here could be useful for the study of LTBI contacts at risk of progression to active TB, without the need of a follow-up study. This is of great utility given the WHO's recommendations of preventive treatment (23), which makes it difficult to perform follow-up studies in untreated LTBI individuals. Our approach, based on the idea that biomarkers of active TB could be used to identify people at risk of progression to active TB, is in agreement with a recent study by Roe et al. (40). In their study, *BATF2*, an active TB-derived biomarker, was used to identify cases of incipient tuberculosis among TB-progressors from Zak's cohort, with promising results. This not only supports the suitability of the approach used in our study, but interestingly, *BATF2* is also amongst the genes up-regulated in our *TB-like* group, supporting a higher risk of progression of these individuals to active TB. In addition to the mechanisms described above, our work provides new information of the events that might correlate with an incipient TB stage. Besides all the above, *TB-like* contacts are also characterized by a higher expression of genes involved in B cell function, T cell regulation and others, that could intervene in *Mtb* infection, such as syndecans and transcobalamin carriers.

Furthermore, the immunological differences between *TB-like* and *NoTB-like* contacts suggested an infectious process taking place in the former. On the one hand, we observed an increase in leukocytes and monocyte proportion, suggested to correlate with risk of progression (41). And higher concentration in *TB-like* contacts of the serum cytokines IL-6, IL-7, TGFα and IL-27 and

the chemokine IP-10, a chemokine proposed as a tool to monitor inflammation and disease activity in TB (42).

While we believe that this approach has a significant potential to be used for better resolution within the broad spectrum of LTBI, we do recognize certain shortcomings of our study. Our cohorts in Spain and Mozambique were relatively small and did not provide us with an opportunity to perform the longitudinal studies to test the predictive power of our model. However, we believe that our findings merit such follow up studies in a larger cohort of LTBI individuals in an endemic TB setting, so that our findings could be validated and this concept potentially harnessed for better management of LTBI.

## Data Availability Statement

The datasets generated for this study can be found in the ArrayExpress database at EMBL-EBI; accession number E-MTAB-7830.

## Ethics Statement

The studies involving human participants were reviewed and approved by Galician Ethics Committee (registry number: 2014/492), National Bioethics Committee for Health of Mozambique (reference number 298/CNBS/2015). The patients/participants provided their written informed consent to participate in this study.

## Author Contributions

LA and ÁG-F conceptualized the study and conceived the project. OE contributed with RNA isolation, study design, and NGS data analysis. EG participated in RNA isolation and results discussions. LA, LC, and LB contributed with the recruitment of the participants and sample collection from the Spanish cohort. ÁP participated in sample processing. TM and CM performed the recruitment and sample processing of Mozambican participants. NF, FF-R, DG-P, MR-J, and HL-F contributed with data analysis and machine learning approach design. RR provided counseling. ÁG-F secured funding and supervised the work. OE wrote the paper with input from all other authors.

## Conflict of Interest

The authors declare that the research was conducted in the absence of any commercial or financial relationships that could be construed as a potential conflict of interest.
