# Supplementary material for: An RNA-seq Based Machine Learning Approach Identifies Latent Tuberculosis Patients With an Active Tuberculosis Profile
Source: Front Immunol. 2020 Jul 14;11:1470. doi: 10.3389/fimmu.2020.01470 (PMC7372107; doi:10.3389/fimmu.2020.01470)
Supplement: Supplementary file 1 [file Data_Sheet_1.docx]

**Fig S1**. **Principal Component Analysis (PCA) of the Spanish (S) and Mozambican (M) cohorts**. A: PCA of the Spanish cohort coloured by group. B: PCA of the Mozambican cohort coloured by group. C: PCA of the Spanish cohort coloured by gender. D: PCA of the Mozambican cohort coloured by gender. The TPM-normalized counts from all gene annotations in the reference genome were used in this analysis. The first two principal components (Dim1 and Dim2) represent the main causes of difference between groups. NoTBI: Uninfected contacts; LTBI: contacts with latent infection; TB: Tuberculosis; F: Female; M: Male.

**Fig S2. Hierarchical Clustering analysis of the active TB patients and their contacts from Mozambique (Test set) based on the expression of the differentially expressed genes derived from the Spanish cohort. (A)** Heatmap based on the 259 DE genes between Active TB and NoTBI contacts. **(B)** Heatmap based on the 133 genes between active TB and LTBI contacts. Each column of the heatmaps represents one sample and each row represents one gene. Both the samples and the genes have been clustered based on the similarity of their expression pattern. The colour of the cells indicates the expression of each gene for the corresponding sample. The input counts matrix for the heatmap was normalized using the DESeq2 Variance stabilizing Transformation (VST) function and rows (i.e. genes) scaled using the pheatmap "scale = row" parameter.


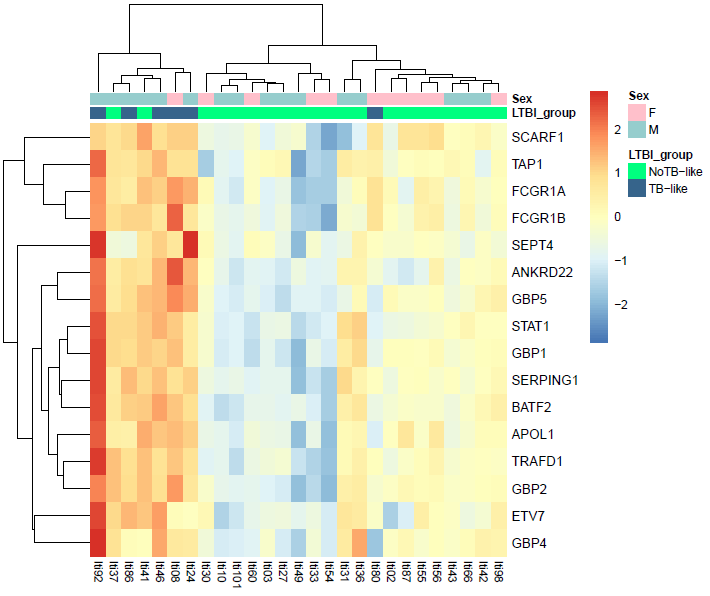


**Fig S3. Hierarchical Clustering analysis of the LTBI subsets based on the expression of the 16-gene signature of risk.** Each column of the heatmap represents one sample and each row represents one gene. Both the samples and the genes have been clustered based on the similarity of their expression pattern. The colour of the cells indicates the expression of each gene for the corresponding sample. The input counts matrix for the heatmap was normalized using the DESeq2 Variance Stabilizing Transformation (VST) function and rows (i.e. genes) scaled using the "scale = row" parameter from the R package “pheatmap”.

***Table S.1.*** ***259-gene signature including the Differentially Expressed (DE) genes between Active TB patients and NoTBI contacts****.*

| **Active TB *vs.* NoTBI** | |  |  |
| --- | --- | --- | --- |
| **Ensembl ID** | **Gene Symbol** | **adj. p value** | **Log2FC** |
| ENSG00000087116 | ADAMTS2 | 1,56965E-12 | 3,656514138 |
| ENSG00000232039 | DEFT1P2 | 0,000153467 | 3,346393572 |
| ENSG00000170439 | METTL7B | 4,81286E-10 | 3,199675281 |
| ENSG00000159189 | C1QC | 3,2231E-14 | 3,010716297 |
| ENSG00000196415 | PRTN3 | 2,32746E-07 | 2,960943941 |
| ENSG00000197561 | ELANE | 1,0107E-07 | 2,816156979 |
| ENSG00000240247 | DEFA1B | 6,10659E-06 | 2,808665615 |
| ENSG00000220586 | TUBBP9 | 0,007833316 | 2,720245599 |
| ENSG00000225492 | GBP1P1 | 5,81822E-09 | 2,718855292 |
| ENSG00000204525 | HLA-C | 0,000579483 | 2,616731598 |
| ENSG00000173369 | C1QB | 2,13266E-12 | 2,531481232 |
| ENSG00000254781 | AC091564 | 0,002330621 | 2,482934419 |
| ENSG00000239839 | DEFA3 | 2,13233E-06 | 2,481953645 |
| ENSG00000204933 | CD177P1 | 0,002250037 | 2,436872477 |
| ENSG00000172232 | AZU1 | 1,56709E-06 | 2,425799877 |
| ENSG00000176532 | PRR15 | 0,001853892 | 2,386097804 |
| ENSG00000137869 | CYP19A1 | 0,001114009 | 2,321472082 |
| ENSG00000005381 | MPO | 2,02583E-07 | 2,315109495 |
| ENSG00000141314 | RHBDL3 | 0,00374866 | 2,291730871 |
| ENSG00000204936 | CD177 | 2,50502E-05 | 2,286958001 |
| ENSG00000122420 | PTGFR | 4,29743E-05 | 2,279118548 |
| ENSG00000102837 | OLFM4 | 0,000473103 | 2,242693599 |
| ENSG00000148346 | LCN2 | 1,74302E-06 | 2,239921821 |
| ENSG00000136160 | EDNRB | 1,58485E-06 | 2,207546801 |
| ENSG00000118113 | MMP8 | 4,48374E-06 | 2,203267421 |
| ENSG00000108950 | FAM20A | 1,70261E-15 | 2,194906369 |
| ENSG00000100448 | CTSG | 0,000100532 | 2,194131315 |
| ENSG00000115884 | SDC1 | 1,36578E-05 | 2,182481814 |
| ENSG00000168062 | BATF2 | 1,76729E-06 | 2,176728118 |
| ENSG00000283706 | PRSS50 | 0,00079257 | 2,135294092 |
| ENSG00000012223 | LTF | 2,94679E-05 | 2,079912669 |
| ENSG00000233105 | AC103563 | 0,002083804 | 2,070978356 |
| ENSG00000101425 | BPI | 6,92443E-06 | 1,954160929 |
| ENSG00000104332 | SFRP1 | 0,000325078 | 1,93430286 |
| ENSG00000118160 | SLC8A2 | 0,005583415 | 1,928056476 |
| ENSG00000086548 | CEACAM6 | 0,000416045 | 1,926187958 |
| ENSG00000173372 | C1QA | 5,26509E-09 | 1,911873843 |
| ENSG00000189221 | MAOA | 0,00017245 | 1,883144933 |
| ENSG00000164821 | DEFA4 | 0,000718458 | 1,876177632 |
| ENSG00000137834 | SMAD6 | 2,02904E-05 | 1,863105776 |
| ENSG00000073734 | ABCB11 | 0,003776386 | 1,818414234 |
| ENSG00000010030 | ETV7 | 2,24078E-05 | 1,816813219 |
| ENSG00000278473 | IGHV3-41 | 0,008261563 | 1,808605794 |
| ENSG00000170801 | HTRA3 | 0,000805172 | 1,792274076 |
| ENSG00000273962 | IGKV2-40 | 0,001169138 | 1,78425316 |
| ENSG00000124469 | CEACAM8 | 0,000645002 | 1,780333994 |
| ENSG00000211896 | IGHG1 | 5,29335E-06 | 1,767442843 |
| ENSG00000163710 | PCOLCE2 | 0,001491427 | 1,762414999 |
| ENSG00000224940 | PRRT4 | 3,33891E-05 | 1,757347013 |
| ENSG00000183856 | IQGAP3 | 7,09975E-05 | 1,741603034 |
| ENSG00000188403 | IGHV1OR15-9 | 0,00196946 | 1,741410934 |
| ENSG00000234745 | HLA-B | 0,002023025 | 1,72692321 |
| ENSG00000203413 | ACTBP6 | 0,004864619 | 1,723153248 |
| ENSG00000164047 | CAMP | 3,07415E-06 | 1,684035855 |
| ENSG00000198959 | TGM2 | 3,13705E-07 | 1,681370958 |
| ENSG00000179869 | ABCA13 | 0,00044477 | 1,646071605 |
| ENSG00000154451 | GBP5 | 1,9136E-05 | 1,644165894 |
| ENSG00000154277 | UCHL1 | 0,007182099 | 1,608940245 |
| ENSG00000211946 | IGHV3-20 | 0,000322554 | 1,588330076 |
| ENSG00000173239 | LIPM | 0,00446243 | 1,584318739 |
| ENSG00000241294 | IGKV2-24 | 5,46047E-06 | 1,582554814 |
| ENSG00000108387 | SEPT4 | 0,000249684 | 1,581375259 |
| ENSG00000166278 | C2 | 8,83372E-06 | 1,580350627 |
| ENSG00000150337 | FCGR1A | 1,091E-05 | 1,573884584 |
| ENSG00000133063 | CHIT1 | 5,8327E-06 | 1,564238821 |
| ENSG00000157554 | ERG | 0,000377174 | 1,56377234 |
| ENSG00000130202 | NECTIN2 | 3,95806E-05 | 1,562676366 |
| ENSG00000124785 | NRN1 | 3,15534E-05 | 1,556800837 |
| ENSG00000275221 | HIST1H2AK | 0,004600855 | 1,547862628 |
| ENSG00000149131 | SERPING1 | 0,000410195 | 1,53958887 |
| ENSG00000147206 | NXF3 | 0,002185952 | 1,527147747 |
| ENSG00000265531 | FCGR1CP | 0,000118685 | 1,510777113 |
| ENSG00000128965 | CHAC1 | 0,00239214 | 1,496574266 |
| ENSG00000065618 | COL17A1 | 0,002311054 | 1,489387903 |
| ENSG00000125968 | ID1 | 0,002860673 | 1,473366692 |
| ENSG00000096006 | CRISP3 | 0,00073933 | 1,470651476 |
| ENSG00000079393 | DUSP13 | 3,9156E-07 | 1,461560204 |
| ENSG00000272636 | DOC2B | 0,000334702 | 1,441886005 |
| ENSG00000175643 | RMI2 | 0,001345021 | 1,430739688 |
| ENSG00000211974 | AC245369 | 0,005846626 | 1,425421779 |
| ENSG00000257017 | HP | 2,94679E-05 | 1,425222757 |
| ENSG00000241755 | IGKV1-9 | 4,06032E-05 | 1,422958411 |
| ENSG00000133106 | EPSTI1 | 0,00019951 | 1,412454324 |
| ENSG00000274576 | IGHV2-70 | 0,000153467 | 1,395729734 |
| ENSG00000169397 | RNASE3 | 0,002381114 | 1,389507553 |
| ENSG00000183347 | GBP6 | 8,55259E-08 | 1,385806602 |
| ENSG00000134827 | TCN1 | 3,01742E-05 | 1,385704362 |
| ENSG00000149516 | MS4A3 | 0,001366669 | 1,375326924 |
| ENSG00000134463 | ECHDC3 | 3,37095E-07 | 1,368185297 |
| ENSG00000211655 | IGLV1-36 | 0,00081707 | 1,366960157 |
| ENSG00000074410 | CA12 | 0,000186529 | 1,350940066 |
| ENSG00000197646 | PDCD1LG2 | 0,007869561 | 1,350887534 |
| ENSG00000122641 | INHBA | 0,009584741 | 1,343030625 |
| ENSG00000142583 | SLC2A5 | 5,00682E-06 | 1,342434395 |
| ENSG00000136634 | IL10 | 8,20374E-05 | 1,339621204 |
| ENSG00000171729 | TMEM51 | 5,7745E-07 | 1,338238784 |
| ENSG00000163958 | ZDHHC19 | 1,70996E-06 | 1,336107896 |
| ENSG00000167680 | SEMA6B | 0,004197483 | 1,324989893 |
| ENSG00000104918 | RETN | 0,000208798 | 1,321910635 |
| ENSG00000253936 | IGHV3-63 | 0,003302945 | 1,314333038 |
| ENSG00000112333 | NR2E1 | 0,004620274 | 1,312297491 |
| ENSG00000255221 | CARD17 | 0,00021213 | 1,310964765 |
| ENSG00000105974 | CAV1 | 0,000136421 | 1,310144013 |
| ENSG00000139572 | GPR84 | 9,89227E-06 | 1,307489444 |
| ENSG00000132170 | PPARG | 7,85307E-05 | 1,301829753 |
| ENSG00000173391 | OLR1 | 0,005200754 | 1,293832488 |
| ENSG00000135424 | ITGA7 | 1,06586E-07 | 1,287501091 |
| ENSG00000162512 | SDC3 | 1,09316E-06 | 1,279164188 |
| ENSG00000214872 | SMTNL1 | 0,001339206 | 1,252044114 |
| ENSG00000242076 | IGKV1-33 | 3,27404E-05 | 1,248118017 |
| ENSG00000116663 | FBXO6 | 1,90251E-07 | 1,245567544 |
| ENSG00000165891 | E2F7 | 0,000201586 | 1,244569795 |
| ENSG00000163568 | AIM2 | 6,99105E-05 | 1,236990746 |
| ENSG00000129173 | E2F8 | 0,000578915 | 1,230544756 |
| ENSG00000211632 | IGKV3D-11 | 0,003336466 | 1,213900093 |
| ENSG00000156113 | KCNMA1 | 0,001607674 | 1,212477484 |
| ENSG00000073737 | DHRS9 | 7,03548E-05 | 1,211746205 |
| ENSG00000275832 | ARHGAP23 | 0,000177807 | 1,206214835 |
| ENSG00000185338 | SOCS1 | 0,001077803 | 1,20430454 |
| ENSG00000136514 | RTP4 | 0,000527635 | 1,203590125 |
| ENSG00000180535 | BHLHA15 | 0,003147262 | 1,196894968 |
| ENSG00000117228 | GBP1 | 0,001647092 | 1,195069151 |
| ENSG00000109321 | AREG | 0,000920049 | 1,190023769 |
| ENSG00000198019 | FCGR1B | 4,67267E-05 | 1,188121378 |
| ENSG00000240382 | IGKV1-17 | 3,61276E-05 | 1,187297625 |
| ENSG00000101187 | SLCO4A1 | 5,19758E-07 | 1,18648424 |
| ENSG00000144668 | ITGA9 | 0,00011696 | 1,186256356 |
| ENSG00000168528 | SERINC2 | 1,39998E-05 | 1,181270683 |
| ENSG00000104970 | KIR3DX1 | 0,000317869 | 1,174830691 |
| ENSG00000254979 | AP000781 | 0,000178823 | 1,169194461 |
| ENSG00000145685 | LHFPL2 | 3,97917E-06 | 1,163256797 |
| ENSG00000170955 | CAVIN3 | 0,001143893 | 1,158826642 |
| ENSG00000168899 | VAMP5 | 3,13705E-07 | 1,142183639 |
| ENSG00000163814 | CDCP1 | 6,65057E-09 | 1,139904786 |
| ENSG00000155659 | VSIG4 | 4,2202E-06 | 1,136740201 |
| ENSG00000174944 | P2RY14 | 0,007912948 | 1,133753072 |
| ENSG00000074660 | SCARF1 | 5,81822E-09 | 1,132516251 |
| ENSG00000270550 | IGHV3-30 | 0,001587241 | 1,131830243 |
| ENSG00000142089 | IFITM3 | 0,003336466 | 1,119674764 |
| ENSG00000219074 | SOD1P1 | 0,00896192 | 1,119054826 |
| ENSG00000185339 | TCN2 | 6,17734E-06 | 1,118623032 |
| ENSG00000198829 | SUCNR1 | 0,000274175 | 1,11738559 |
| ENSG00000124145 | SDC4 | 4,86139E-05 | 1,116882837 |
| ENSG00000128322 | IGLL1 | 0,005629014 | 1,113331312 |
| ENSG00000089685 | BIRC5 | 3,98149E-05 | 1,112775918 |
| ENSG00000211653 | IGLV1-40 | 1,32239E-05 | 1,112433861 |
| ENSG00000174705 | SH3PXD2B | 1,7029E-05 | 1,109230916 |
| ENSG00000104808 | DHDH | 7,06305E-06 | 1,10622863 |
| ENSG00000241351 | IGKV3-11 | 3,57225E-05 | 1,102509051 |
| ENSG00000272398 | CD24 | 0,003976577 | 1,095268777 |
| ENSG00000211677 | IGLC2 | 0,000164215 | 1,092969885 |
| ENSG00000185909 | KLHDC8B | 9,213E-09 | 1,089726049 |
| ENSG00000184838 | PRR16 | 0,000271736 | 1,077212003 |
| ENSG00000092067 | CEBPE | 0,000643016 | 1,077112517 |
| ENSG00000135094 | SDS | 0,000211811 | 1,076170947 |
| ENSG00000079215 | SLC1A3 | 0,001550664 | 1,070757079 |
| ENSG00000176845 | METRNL | 5,65166E-05 | 1,066245581 |
| ENSG00000211663 | IGLV3-19 | 3,2451E-05 | 1,063732297 |
| ENSG00000224373 | IGHV4-59 | 0,000165991 | 1,060618893 |
| ENSG00000107566 | ERLIN1 | 1,24768E-07 | 1,059222533 |
| ENSG00000248208 | WDR45P1 | 0,001997304 | 1,057574441 |
| ENSG00000152229 | PSTPIP2 | 1,13171E-05 | 1,056469777 |
| ENSG00000064270 | ATP2C2 | 0,001176249 | 1,052752503 |
| ENSG00000078399 | HOXA9 | 0,000291998 | 1,052566139 |
| ENSG00000156587 | UBE2L6 | 1,23497E-05 | 1,051293837 |
| ENSG00000211592 | IGKC | 5,60634E-05 | 1,049742832 |
| ENSG00000204345 | CD300LD | 0,005172053 | 1,044321092 |
| ENSG00000167207 | NOD2 | 2,88884E-07 | 1,036986699 |
| ENSG00000121933 | TMIGD3 | 0,004637133 | 1,036824952 |
| ENSG00000211895 | IGHA1 | 0,001301007 | 1,032325264 |
| ENSG00000119686 | FLVCR2 | 1,9825E-08 | 1,03157007 |
| ENSG00000139410 | SDSL | 1,091E-05 | 1,030850327 |
| ENSG00000211640 | IGLV6-57 | 0,000453579 | 1,029860803 |
| ENSG00000254827 | SLC22A18AS | 1,02294E-05 | 1,029551875 |
| ENSG00000150760 | DOCK1 | 4,90555E-06 | 1,028518357 |
| ENSG00000112984 | KIF20A | 0,003371272 | 1,024923868 |
| ENSG00000038945 | MSR1 | 0,000515565 | 1,024662756 |
| ENSG00000170476 | MZB1 | 5,43925E-05 | 1,011279764 |
| ENSG00000165685 | TMEM52B | 0,000382986 | 1,009501022 |
| ENSG00000211658 | IGLV3-27 | 0,00947824 | 1,00668421 |
| ENSG00000160180 | TFF3 | 0,009080092 | 1,00215731 |
| ENSG00000211596 | IGKJ2 | 0,002302288 | 1,002002761 |
| ENSG00000151025 | GPR158 | 0,000139287 | 1,001357856 |
| ENSG00000169031 | COL4A3 | 8,49418E-05 | -1,00799412 |
| ENSG00000164742 | ADCY1 | 0,005348759 | -1,009981128 |
| ENSG00000236946 | HNRNPA1P70 | 0,00117123 | -1,012168749 |
| ENSG00000101680 | LAMA1 | 0,002179709 | -1,020802141 |
| ENSG00000099769 | IGFALS | 0,00627983 | -1,023925486 |
| ENSG00000002745 | WNT16 | 0,001131941 | -1,026065046 |
| ENSG00000268225 | AC010487 | 0,002786723 | -1,032853799 |
| ENSG00000136244 | IL6 | 0,005687631 | -1,040321679 |
| ENSG00000162630 | B3GALT2 | 0,002936683 | -1,041224694 |
| ENSG00000213557 | AC068050 | 0,000168093 | -1,043419367 |
| ENSG00000162571 | TTLL10 | 0,000570657 | -1,046766051 |
| ENSG00000211882 | TRAJ7 | 0,009311484 | -1,048012449 |
| ENSG00000091129 | NRCAM | 0,001871504 | -1,054156912 |
| ENSG00000275395 | FCGBP | 6,92443E-06 | -1,054797107 |
| ENSG00000258732 | AC025884 | 0,000349439 | -1,066344641 |
| ENSG00000144290 | SLC4A10 | 0,000790273 | -1,066809565 |
| ENSG00000225526 | MKRN2OS | 9,43872E-05 | -1,069447539 |
| ENSG00000126838 | PZP | 0,001595696 | -1,070745653 |
| ENSG00000262209 | PCDHGB3 | 0,00700758 | -1,075609714 |
| ENSG00000206077 | ZDHHC11B | 8,25477E-05 | -1,088730613 |
| ENSG00000214575 | CPEB1 | 0,000732745 | -1,09006073 |
| ENSG00000164283 | ESM1 | 0,004258658 | -1,090503028 |
| ENSG00000275772 | AC244157 | 6,88406E-05 | -1,095400417 |
| ENSG00000221970 | OR2A1 | 0,00113392 | -1,104292291 |
| ENSG00000211866 | TRAJ23 | 0,001974786 | -1,10463512 |
| ENSG00000185689 | C6orf201 | 0,001867353 | -1,106424195 |
| ENSG00000262187 | AC137800 | 0,00255298 | -1,108196139 |
| ENSG00000152779 | SLC16A12 | 0,009349948 | -1,123855496 |
| ENSG00000134532 | SOX5 | 0,00116269 | -1,139538599 |
| ENSG00000211886 | TRAJ3 | 0,000512546 | -1,143274475 |
| ENSG00000120057 | SFRP5 | 0,001835983 | -1,153750516 |
| ENSG00000163485 | ADORA1 | 0,008307075 | -1,154430259 |
| ENSG00000158246 | FAM46B | 0,005778803 | -1,15581042 |
| ENSG00000185818 | NAT8L | 0,00647639 | -1,157489431 |
| ENSG00000107018 | RLN1 | 0,001883526 | -1,158335756 |
| ENSG00000216588 | IGSF23 | 0,00103795 | -1,166025334 |
| ENSG00000171798 | KNDC1 | 0,000766376 | -1,175289178 |
| ENSG00000174469 | CNTNAP2 | 1,17997E-05 | -1,199536902 |
| ENSG00000187999 | HNRNPA1P61 | 0,002228978 | -1,212533857 |
| ENSG00000283041 | AC008038 | 0,004564605 | -1,218568937 |
| ENSG00000242636 | RPL21P129 | 0,00754473 | -1,230849164 |
| ENSG00000164744 | SUN3 | 0,004683327 | -1,273521408 |
| ENSG00000135373 | EHF | 0,006369571 | -1,302200794 |
| ENSG00000244050 | DEFB109F | 3,09807E-05 | -1,309480832 |
| ENSG00000249454 | GZMAP1 | 0,006762613 | -1,369303732 |
| ENSG00000189367 | KIAA0408 | 0,000808122 | -1,423730842 |
| ENSG00000257752 | AC091516 | 0,006184003 | -1,4298976 |
| ENSG00000164746 | C7orf57 | 0,005413541 | -1,455259585 |
| ENSG00000121743 | GJA3 | 6,6784E-05 | -1,532158161 |
| ENSG00000168032 | ENTPD3 | 0,001143893 | -1,55584217 |
| ENSG00000232608 | TIMM9P2 | 0,005733992 | -1,564719296 |
| ENSG00000256713 | PGA5 | 0,004321505 | -1,597132019 |
| ENSG00000273423 | OR13I1P | 0,005634936 | -1,611830179 |
| ENSG00000229859 | PGA3 | 3,02247E-05 | -1,628523215 |
| ENSG00000176771 | NCKAP5 | 0,002103773 | -1,659938117 |
| ENSG00000146250 | PRSS35 | 0,000868762 | -1,712379164 |
| ENSG00000049247 | UTS2 | 0,000186529 | -1,713642282 |
| ENSG00000241404 | EGFL8 | 0,003311369 | -1,820338523 |
| ENSG00000172476 | RAB40A | 0,003570127 | -1,833184114 |
| ENSG00000275801 | AL121985 | 0,00109397 | -1,840502914 |
| ENSG00000232032 | AC079781 | 0,009270325 | -1,860916181 |
| ENSG00000231344 | AL020997 | 0,005737851 | -1,877287032 |
| ENSG00000180574 | AC068775 | 0,00396542 | -1,889661612 |
| ENSG00000130720 | FIBCD1 | 0,007640702 | -1,903065112 |
| ENSG00000109163 | GNRHR | 0,000961937 | -1,90816216 |
| ENSG00000256188 | TAS2R30 | 0,006095386 | -1,984548468 |
| ENSG00000214351 | OR1X5P | 0,00457851 | -2,048941409 |
| ENSG00000115353 | TACR1 | 0,0014269 | -2,084371692 |
| ENSG00000146477 | SLC22A3 | 0,000962328 | -2,092789755 |
| ENSG00000141579 | ZNF750 | 0,000425189 | -2,130978193 |
| ENSG00000231831 | MTHFD1P1 | 0,003902389 | -2,267331686 |
| ENSG00000204583 | LRCOL1 | 0,002063568 | -2,305020624 |
| ENSG00000163873 | GRIK3 | 0,002059679 | -2,338769478 |
| ENSG00000248340 | AC106047 | 0,004382937 | -2,34330096 |
| ENSG00000233877 | AL606517 | 0,001371644 | -2,375759889 |
| ENSG00000224886 | AL132656 | 0,000229239 | -2,713784649 |

*Genes are identified by their Gene symbol and the corresponding Ensembl ID. All genes included in the list presented significant differences (adjusted p value <0.01) between the groups being compared and an absolute Log2 fold change <1. Genes with Log2CF > 1 are up-regulated and Lof2FC < -1 down-regulated in TB. Differential expression was calculated using the R package DESeq2 with the default parameters (p-values were calculated by the Wald test and corrected for multiple testing using the Benjamini and Hochberg method).*

***Table S.2.*** ***133-gene signature including the Differentially Expressed (DE) genes between Active TB patients and LTBI contacts****.*

| **Active TB *vs.* LTBI** |  |  |  |
| --- | --- | --- | --- |
| **Ensembl ID** | **Gene Symbol** | **adj. p value** | **Log2FC** |
| ENSG00000249119 | MTND6P4 | 3,14077E-06 | 3,438262468 |
| ENSG00000087116 | ADAMTS2 | 1,35541E-05 | 3,208000726 |
| ENSG00000196415 | PRTN3 | 9,88302E-05 | 2,931234173 |
| ENSG00000204498 | NFKBIL1 | 0,000142164 | 2,929354032 |
| ENSG00000170439 | METTL7B | 3,40063E-05 | 2,912194366 |
| ENSG00000204525 | HLA-C | 0,00277091 | 2,72871966 |
| ENSG00000239839 | DEFA3 | 0,000240503 | 2,618131608 |
| ENSG00000197561 | ELANE | 9,88302E-05 | 2,571014927 |
| ENSG00000225492 | GBP1P1 | 0,000212364 | 2,198787569 |
| ENSG00000159189 | C1QC | 5,57133E-05 | 2,144061017 |
| ENSG00000172232 | AZU1 | 0,000597464 | 2,143507104 |
| ENSG00000122420 | PTGFR | 0,000512276 | 2,135324135 |
| ENSG00000100448 | CTSG | 0,001661992 | 2,116012574 |
| ENSG00000152766 | ANKRD22 | 4,09996E-05 | 2,110067614 |
| ENSG00000240247 | DEFA1B | 0,003644118 | 2,03040891 |
| ENSG00000164821 | DEFA4 | 0,001996923 | 2,008317033 |
| ENSG00000168062 | BATF2 | 5,57133E-05 | 1,997230484 |
| ENSG00000005381 | MPO | 0,000495645 | 1,963512468 |
| ENSG00000234745 | HLA-B | 0,003416126 | 1,946624882 |
| ENSG00000224940 | PRRT4 | 2,0884E-05 | 1,908261882 |
| ENSG00000183856 | IQGAP3 | 9,24079E-05 | 1,887457919 |
| ENSG00000108950 | FAM20A | 1,35541E-05 | 1,843783674 |
| ENSG00000108387 | SEPT4 | 0,000115623 | 1,829944814 |
| ENSG00000010030 | ETV7 | 9,24079E-05 | 1,827267699 |
| ENSG00000173239 | LIPM | 0,001655652 | 1,804954444 |
| ENSG00000124469 | CEACAM8 | 0,003536039 | 1,795863104 |
| ENSG00000175643 | RMI2 | 0,000503159 | 1,793199695 |
| ENSG00000179869 | ABCA13 | 0,001401728 | 1,759052843 |
| ENSG00000148346 | LCN2 | 0,003829192 | 1,755526781 |
| ENSG00000104972 | LILRB1 | 0,001329198 | 1,745039805 |
| ENSG00000173320 | STOX2 | 0,002606112 | 1,737532226 |
| ENSG00000257017 | HP | 9,88302E-05 | 1,730038265 |
| ENSG00000065618 | COL17A1 | 0,002260641 | 1,713488469 |
| ENSG00000012223 | LTF | 0,007125273 | 1,711873668 |
| ENSG00000118113 | MMP8 | 0,006813097 | 1,706767909 |
| ENSG00000157554 | ERG | 0,000589568 | 1,684897338 |
| ENSG00000074410 | CA12 | 0,000512276 | 1,672441617 |
| ENSG00000197582 | GPX1P1 | 0,003180189 | 1,670346507 |
| ENSG00000124785 | NRN1 | 0,000853764 | 1,652396714 |
| ENSG00000173369 | C1QB | 0,001088308 | 1,639237354 |
| ENSG00000122641 | INHBA | 0,005043275 | 1,582998033 |
| ENSG00000115155 | OTOF | 0,001756064 | 1,555841031 |
| ENSG00000101425 | BPI | 0,00748428 | 1,548292821 |
| ENSG00000133063 | CHIT1 | 0,00050639 | 1,536590181 |
| ENSG00000255221 | CARD17 | 5,57133E-05 | 1,525197527 |
| ENSG00000139572 | GPR84 | 2,63216E-05 | 1,506964341 |
| ENSG00000154451 | GBP5 | 0,001461818 | 1,474067548 |
| ENSG00000170955 | CAVIN3 | 0,001153924 | 1,460790558 |
| ENSG00000174944 | P2RY14 | 0,000597464 | 1,460648079 |
| ENSG00000149131 | SERPING1 | 0,001680723 | 1,450279664 |
| ENSG00000164047 | CAMP | 0,002327631 | 1,429273363 |
| ENSG00000136160 | EDNRB | 0,006777681 | 1,418312619 |
| ENSG00000173391 | OLR1 | 0,008459038 | 1,411816533 |
| ENSG00000167680 | SEMA6B | 0,009832226 | 1,41170974 |
| ENSG00000198959 | TGM2 | 0,000595092 | 1,401890306 |
| ENSG00000170091 | HGNC:24955 | 0,002407174 | 1,370054904 |
| ENSG00000104918 | RETN | 0,002300701 | 1,365691592 |
| ENSG00000211896 | IGHG1 | 0,008660075 | 1,357444706 |
| ENSG00000213722 | DDAH2 | 0,007143417 | 1,353926525 |
| ENSG00000134827 | TCN1 | 0,001871764 | 1,344821876 |
| ENSG00000073737 | DHRS9 | 0,000597464 | 1,331506198 |
| ENSG00000079215 | SLC1A3 | 0,00114957 | 1,330100178 |
| ENSG00000167105 | TMEM92 | 0,000431467 | 1,306528129 |
| ENSG00000173372 | C1QA | 0,002275206 | 1,304096314 |
| ENSG00000150337 | FCGR1A | 0,002111357 | 1,299649151 |
| ENSG00000163568 | AIM2 | 0,000129728 | 1,294181135 |
| ENSG00000168528 | SERINC2 | 9,88302E-05 | 1,280147544 |
| ENSG00000196565 | HBG2 | 0,000941207 | 1,279907806 |
| ENSG00000204305 | AGER | 0,005210471 | 1,277497051 |
| ENSG00000011201 | ANOS1 | 0,001911237 | 1,274039107 |
| ENSG00000079393 | DUSP13 | 0,000376136 | 1,260632061 |
| ENSG00000166278 | C2 | 0,002978028 | 1,225669362 |
| ENSG00000120217 | CD274 | 0,002276984 | 1,221096132 |
| ENSG00000158163 | DZIP1L | 0,001447362 | 1,216743718 |
| ENSG00000185338 | SOCS1 | 0,000880208 | 1,2150867 |
| ENSG00000142583 | SLC2A5 | 0,001056361 | 1,212574193 |
| ENSG00000198829 | SUCNR1 | 0,000655701 | 1,196884075 |
| ENSG00000136514 | RTP4 | 0,003233098 | 1,194628448 |
| ENSG00000214872 | SMTNL1 | 0,002425222 | 1,19418716 |
| ENSG00000117228 | GBP1 | 0,001772202 | 1,189648879 |
| ENSG00000186818 | LILRB4 | 0,003486001 | 1,18903986 |
| ENSG00000203812 | HIST2H2AA3 | 0,000106586 | 1,170264165 |
| ENSG00000211596 | IGKJ2 | 0,003610197 | 1,167513626 |
| ENSG00000165891 | E2F7 | 0,002378891 | 1,163916235 |
| ENSG00000157168 | NRG1 | 0,000263816 | 1,160918915 |
| ENSG00000197506 | SLC28A3 | 0,00566009 | 1,160350036 |
| ENSG00000134463 | ECHDC3 | 0,00157716 | 1,139906151 |
| ENSG00000196747 | HIST1H2AI | 0,002209195 | 1,138151052 |
| ENSG00000271304 | AL133507 | 0,007979561 | 1,126199386 |
| ENSG00000183347 | GBP6 | 0,002395442 | 1,115628561 |
| ENSG00000116663 | FBXO6 | 9,88302E-05 | 1,115159407 |
| ENSG00000180878 | C11orf42 | 0,003178885 | 1,111664129 |
| ENSG00000211597 | IGKJ1 | 0,007360585 | 1,107851953 |
| ENSG00000118804 | STBD1 | 0,001988424 | 1,103198742 |
| ENSG00000078399 | HOXA9 | 0,000744973 | 1,096692182 |
| ENSG00000100985 | MMP9 | 0,002875846 | 1,096669278 |
| ENSG00000092067 | CEBPE | 0,001719186 | 1,095608098 |
| ENSG00000185909 | KLHDC8B | 1,04798E-05 | 1,073007842 |
| ENSG00000145685 | LHFPL2 | 0,000264482 | 1,048791503 |
| ENSG00000198216 | CACNA1E | 0,000604505 | 1,045930653 |
| ENSG00000165685 | TMEM52B | 0,001641145 | 1,025372127 |
| ENSG00000197632 | SERPINB2 | 0,007340172 | 1,024619692 |
| ENSG00000172159 | FRMD3 | 0,000897685 | 1,02033345 |
| ENSG00000155659 | VSIG4 | 0,00161518 | 1,020212775 |
| ENSG00000186583 | SPATC1 | 0,003222299 | 1,009902513 |
| ENSG00000162512 | SDC3 | 0,0029404 | 1,005229104 |
| ENSG00000170848 | PSG6 | 0,005713397 | 1,003521497 |
| ENSG00000102010 | BMX | 0,000880208 | 1,001313735 |
| ENSG00000239998 | LILRA2 | 0,002781952 | 1,000882165 |
| ENSG00000165731 | RET | 0,001993654 | -1,033635487 |
| ENSG00000149256 | TENM4 | 0,002474708 | -1,058250459 |
| ENSG00000130635 | COL5A1 | 0,006067009 | -1,082708533 |
| ENSG00000227212 | PFN1P6 | 0,005439046 | -1,096962048 |
| ENSG00000275772 | AC244157 | 0,002829605 | -1,116169312 |
| ENSG00000198796 | ALPK2 | 0,001655652 | -1,167140788 |
| ENSG00000185483 | ROR1 | 0,005329816 | -1,173137845 |
| ENSG00000226321 | CROCC2 | 0,002157449 | -1,173283388 |
| ENSG00000244050 | DEFB109F | 0,002874926 | -1,186301058 |
| ENSG00000025423 | HSD17B6 | 0,004576092 | -1,199752648 |
| ENSG00000002745 | WNT16 | 0,002060824 | -1,249785005 |
| ENSG00000150625 | GPM6A | 0,000744973 | -1,276596397 |
| ENSG00000197353 | LYPD2 | 0,009487561 | -1,317651319 |
| ENSG00000241134 | BET1P1 | 0,007924208 | -1,319804789 |
| ENSG00000229859 | PGA3 | 0,004983615 | -1,332134496 |
| ENSG00000164283 | ESM1 | 0,002575365 | -1,337124863 |
| ENSG00000236946 | HNRNPA1P70 | 0,000293572 | -1,36059202 |
| ENSG00000189367 | KIAA0408 | 0,000943453 | -1,412276193 |
| ENSG00000006468 | ETV1 | 0,001565788 | -1,418501681 |
| ENSG00000171798 | KNDC1 | 0,00139985 | -1,491297549 |
| ENSG00000174473 | GALNTL6 | 0,00803163 | -1,497380348 |
| ENSG00000258732 | AC025884 | 5,57133E-05 | -1,541861561 |
| ENSG00000169031 | COL4A3 | 5,57133E-05 | -1,666578932 |
| ENSG00000172987 | HPSE2 | 0,000597464 | -2,210898842 |

*Genes are identified by their Gene symbol and the corresponding Ensembl ID. All genes included in the list presented significant differences (adjusted p value <0.01) between the groups being compared and an absolute Log2 fold change <1. Genes with Log2CF > 1 are up-regulated and Lof2FC < -1 down-regulated in TB. Differential expression was calculated using the R package DESeq2 with the default parameters (p-values were calculated by the Wald test and corrected for multiple testing using the Benjamini and Hochberg method).*

***Table S.3.*** ***Reactome Pathway enrichment analysis of the 259-gene signature differentiating Active TB vs. NoTBI****.*

| **Pathway enrichment: Active TB vs. NoTBI** | | | | |
| --- | --- | --- | --- | --- |
| **Pathway ID** | **Pathway Description** | **#Genes** | **p.adjust** | **gene ID (genes in the input list)** |
| R-HSA-6798695 | Neutrophil degranulation | 29 | 1,97E-10 | OLR1, GPR84, RETN, SLC2A5, MS4A3, TCN1, RNASE3, HP, CRISP3, CHIT1, ABCA13, CAMP, HLA-B, CEACAM8, DEFA4, CEACAM6, BPI, LTF, CTSG, MMP8, LCN2, OLFM4, CD177, MPO, AZU1, HLA-C, DEFA1B, ELANE, PRTN3 |
| R-HSA-6803157 | Antimicrobial peptides | 11 | 4,83E-06 | RNASE3, CAMP, DEFA4, BPI, LTF, CTSG, LCN2, DEFA3, DEFA1B, ELANE, PRTN3 |
| R-HSA-1474244 | Extracellular matrix organization | 15 | 5,13E-04 | LAMA1, COL4A3, SDC4, ITGA9, SDC3, ITGA7, COL17A1, PCOLCE2, CEACAM8, CEACAM6, SDC1, CTSG, MMP8, ELANE, ADAMTS2 |
| R-HSA-877300 | Interferon gamma signaling | 8 | 1,54E-03 | FCGR1B, GBP1, SOCS1, GBP6, FCGR1A, GBP5, HLA-B, HLA-C |
| R-HSA-977606 | Regulation of Complement cascade | 6 | 1,63E-03 | SERPING1, C2, C1QA, C1QB, ELANE, C1QC |
| R-HSA-202733 | Cell surface interactions at the vascular wall | 9 | 2,89E-03 | IGLL1, SDC4, SDC3, OLR1, CAV1, CEACAM8, CEACAM6, SDC1, CD177 |
| R-HSA-166658 | Complement cascade | 6 | 3,93E-03 | SERPING1, C2, C1QA, C1QB, ELANE, C1QC |
| R-HSA-913531 | Interferon Signaling | 10 | 6,65E-03 | UBE2L6, IFITM3, FCGR1B, GBP1, SOCS1, GBP6, FCGR1A, GBP5, HLA-B, HLA-C |
| R-HSA-166663 | Initial triggering of complement | 4 | 6,65E-03 | C2, C1QA, C1QB, C1QC |
| R-HSA-1462054 | Alpha-defensins | 3 | 7,59E-03 | DEFA4, DEFA3, DEFA1B |
| R-HSA-166786 | Creation of C4 and C2 activators | 3 | 1,71E-02 | C1QA, C1QB, C1QC |
| R-HSA-3656237 | Defective EXT2 causes exostoses 2 | 3 | 1,71E-02 | SDC4, SDC3, SDC1 |
| R-HSA-3656253 | Defective EXT1 causes exostoses 1, TRPS2 and CHDS | 3 | 1,71E-02 | SDC4, SDC3, SDC1 |
| R-HSA-3000171 | Non-integrin membrane-ECM interactions | 5 | 2,03E-02 | LAMA1, COL4A3, SDC4, SDC3, SDC1 |
| R-HSA-3560783 | Defective B4GALT7 causes EDS, progeroid type | 3 | 3,88E-02 | SDC4, SDC3, SDC1 |
| R-HSA-3560801 | Defective B3GAT3 causes JDSSDHD | 3 | 3,88E-02 | SDC4, SDC3, SDC1 |
| R-HSA-4420332 | Defective B3GALT6 causes EDSP2 and SEMDJL1 | 3 | 3,88E-02 | SDC4, SDC3, SDC1 |
| R-HSA-6785807 | Interleukin-4 and Interleukin-13 signaling | 6 | 4,38E-02 | IL6, BIRC5, SOCS1, IL10, MAOA, LCN2 |
| R-HSA-2024096 | HS-GAG degradation | 3 | 4,60E-02 | SDC4, SDC3, SDC1 |

***Table S.4.*** ***Reactome Pathway enrichment analysis of the 133-gene signature differentiating Active TB vs. NoTBI****.*

| **Pathway enrichment: Active TB vs. LTBI** | | | | |
| --- | --- | --- | --- | --- |
| **Pathway ID** | **Pathway Description** | **#Genes** | **p.adjust** | **gene ID (genes in the input list)** |
| R-HSA-6798695 | Neutrophil degranulation | 25 | 5,43E-12 | MMP9, STBD1, SLC2A5, TCN1, RETN, OLR1, CAMP, GPR84, CHIT1, BPI, MMP8, LTF, HP, LCN2, ABCA13, CEACAM8, HLA-B, MPO, DEFA4, DEFA1B, CTSG, AZU1, ELANE, HLA-C, PRTN3 |
| R-HSA-6803157 | Antimicrobial peptides | 10 | 6,30E-07 | CAMP, BPI, LTF, LCN2, DEFA4, DEFA1B, CTSG, ELANE, DEFA3, PRTN3 |
| R-HSA-977606 | Regulation of Complement cascade | 6 | 1,74E-04 | C2, C1QA, SERPING1, C1QB, C1QC, ELANE |
| R-HSA-166658 | Complement cascade | 6 | 4,60E-04 | C2, C1QA, SERPING1, C1QB, C1QC, ELANE |
| R-HSA-877300 | Interferon gamma signaling | 7 | 4,75E-04 | GBP6, GBP1, SOCS1, FCGR1A, GBP5, HLA-B, HLA-C |
| R-HSA-1442490 | Collagen degradation | 6 | 5,46E-04 | COL4A3, COL5A1, MMP9, MMP8, COL17A1, ELANE |
| R-HSA-166663 | Initial triggering of complement | 4 | 1,25E-03 | C2, C1QA, C1QB, C1QC |
| R-HSA-1462054 | Alpha-defensins | 3 | 2,09E-03 | DEFA4, DEFA1B, DEFA3 |
| R-HSA-198933 | Immunoregulatory interactions between a Lymphoid and a non-Lymphoid cell | 7 | 2,75E-03 | LILRA2, LILRB4, FCGR1A, COL17A1, LILRB1, HLA-B, HLA-C |
| R-HSA-1474228 | Degradation of the extracellular matrix | 7 | 3,25E-03 | COL4A3, COL5A1, MMP9, MMP8, COL17A1, CTSG, ELANE |
| R-HSA-1592389 | Activation of Matrix Metalloproteinases | 4 | 3,25E-03 | MMP9, MMP8, CTSG, ELANE |
| R-HSA-1474244 | Extracellular matrix organization | 10 | 3,25E-03 | COL4A3, COL5A1, SDC3, MMP9, MMP8, COL17A1, CEACAM8, CTSG, ELANE, ADAMTS2 |
| R-HSA-166786 | Creation of C4 and C2 activators | 3 | 3,81E-03 | C1QA, C1QB, C1QC |
| R-HSA-1474290 | Collagen formation | 5 | 1,56E-02 | COL4A3, COL5A1, MMP9, COL17A1, ADAMTS2 |
| R-HSA-913531 | Interferon Signaling | 7 | 1,90E-02 | GBP6, GBP1, SOCS1, FCGR1A, GBP5, HLA-B, HLA-C |
| R-HSA-2022090 | Assembly of collagen fibrils and other multimeric structures | 4 | 2,57E-02 | COL4A3, COL5A1, MMP9, COL17A1 |
| R-HSA-1650814 | Collagen biosynthesis and modifying enzymes | 4 | 3,43E-02 | COL4A3, COL5A1, COL17A1, ADAMTS2 |

**Table S.5: Performance of the Random Forest classification model on the Test set (Mozambique)**

| **Performance in Test set** | |
| --- | --- |
| **Correctly classified instances** | 89% |
| **Kappa** | 0,69 |
| **Specificity** | 88,90% |
| **Sensitivity** | 89,20% |
|  |  |

***Table S.6.*** ***150-gene signature including the Differentially Expressed (DE) genes between TB-like and NoTB-like LTBI contacts****.*

| **TB-like *vs.* NoTB-like** |  |  |  |
| --- | --- | --- | --- |
| **Ensembl ID** | **Gene Symbol** | **adj. p value** | **Log2FC** |
| ENSG00000204619 | PPP1R11 | 2,067E-10 | -23,4230736 |
| ENSG00000276345 | AC004556 | 3,0631E-06 | -22,3596096 |
| ENSG00000074803 | SLC12A1 | 0,00855509 | -3,53198028 |
| ENSG00000270249 | AC093668 | 0,0310106 | -3,2311509 |
| ENSG00000267459 | AC006116 | 0,01850385 | -3,18433687 |
| ENSG00000226981 | ABHD17AP6 | 0,02076062 | -2,23618325 |
| ENSG00000261208 | AL365475 | 0,00336806 | -2,17850428 |
| ENSG00000215559 | ANKRD20A11P | 0,02906322 | -2,13990727 |
| ENSG00000255526 | NEDD8-MDP1 | 0,04956699 | -2,04124654 |
| ENSG00000180447 | GAS1 | 0,02812783 | -1,94344892 |
| ENSG00000116833 | NR5A2 | 0,01534385 | -1,90450199 |
| ENSG00000215493 | AC007731 | 0,0272186 | -1,68253964 |
| ENSG00000154188 | ANGPT1 | 0,00716235 | -1,63303332 |
| ENSG00000224427 | AP000281 | 0,01850385 | -1,50562161 |
| ENSG00000174348 | PODN | 0,01688748 | -1,46090216 |
| ENSG00000176723 | ZNF843 | 0,01059113 | -1,40926004 |
| ENSG00000182389 | CACNB4 | 0,01179734 | -1,27790498 |
| ENSG00000112414 | ADGRG6 | 0,02440293 | -1,20762609 |
| ENSG00000279058 | AGAP14 | 0,0288248 | -1,18608068 |
| ENSG00000183542 | KLRC4 | 0,04860774 | -1,16811398 |
| ENSG00000255819 | KLRC4-KLRK1 | 0,00413399 | -1,15096667 |
| ENSG00000135525 | MAP7 | 0,02471827 | -1,10372041 |
| ENSG00000129167 | TPH1 | 0,01058469 | -1,09847193 |
| ENSG00000197057 | DTHD1 | 0,00011952 | -1,03922112 |
| ENSG00000187984 | ANKRD19P | 0,00028164 | -1,02679394 |
| ENSG00000178075 | GRAMD1C | 0,0237425 | -1,00896775 |
| ENSG00000166851 | PLK1 | 0,01535092 | 1,0007542 |
| ENSG00000135047 | CTSL | 0,01832765 | 1,00377245 |
| ENSG00000099860 | GADD45B | 0,01688748 | 1,00691523 |
| ENSG00000124762 | CDKN1A | 0,03124805 | 1,00940767 |
| ENSG00000188282 | RUFY4 | 0,01422814 | 1,02358627 |
| ENSG00000171848 | RRM2 | 0,01677234 | 1,03010514 |
| ENSG00000177989 | ODF3B | 0,0067429 | 1,03760508 |
| ENSG00000221963 | APOL6 | 0,02204647 | 1,04937409 |
| ENSG00000172159 | FRMD3 | 0,04602867 | 1,05544939 |
| ENSG00000197272 | IL27 | 0,00038159 | 1,06345441 |
| ENSG00000185885 | IFITM1 | 0,00209837 | 1,07672219 |
| ENSG00000134247 | PTGFRN | 0,00057705 | 1,08930875 |
| ENSG00000130489 | SCO2 | 0,0194635 | 1,09416777 |
| ENSG00000141664 | ZCCHC2 | 0,00748121 | 1,09835991 |
| ENSG00000116663 | FBXO6 | 0,00209837 | 1,12343242 |
| ENSG00000125148 | MT2A | 0,03355569 | 1,12595381 |
| ENSG00000174705 | SH3PXD2B | 0,03412456 | 1,12787665 |
| ENSG00000140464 | PML | 0,00060289 | 1,13090089 |
| ENSG00000089685 | BIRC5 | 0,02471827 | 1,13268517 |
| ENSG00000188313 | PLSCR1 | 0,0191164 | 1,13665045 |
| ENSG00000204323 | SMIM5 | 0,04159358 | 1,14571351 |
| ENSG00000068079 | IFI35 | 0,00549598 | 1,15492002 |
| ENSG00000169679 | BUB1 | 0,00206319 | 1,15505614 |
| ENSG00000236567 | TCF3P1 | 0,0437625 | 1,16545679 |
| ENSG00000105971 | CAV2 | 0,00360982 | 1,17141525 |
| ENSG00000229754 | CXCR2P1 | 0,02927228 | 1,18742583 |
| ENSG00000176170 | SPHK1 | 0,0207145 | 1,19498052 |
| ENSG00000148926 | ADM | 0,03904432 | 1,2083566 |
| ENSG00000177706 | FAM20C | 2,0122E-05 | 1,22051715 |
| ENSG00000255221 | CARD17 | 0,03391805 | 1,22129206 |
| ENSG00000101057 | MYBL2 | 0,01641695 | 1,23359971 |
| ENSG00000137628 | DDX60 | 0,0391796 | 1,24638552 |
| ENSG00000132465 | JCHAIN | 0,02940712 | 1,25436038 |
| ENSG00000139832 | RAB20 | 0,00855348 | 1,2583302 |
| ENSG00000119686 | FLVCR2 | 3,5111E-06 | 1,30044032 |
| ENSG00000108950 | FAM20A | 0,00481334 | 1,31992926 |
| ENSG00000254709 | IGLL5 | 0,0348556 | 1,33382121 |
| ENSG00000078081 | LAMP3 | 0,03032517 | 1,34706194 |
| ENSG00000211949 | IGHV3-23 | 0,01116378 | 1,35016279 |
| ENSG00000185339 | TCN2 | 0,00012432 | 1,35419564 |
| ENSG00000239264 | TXNDC5 | 0,00060289 | 1,43874512 |
| ENSG00000240382 | IGKV1-17 | 0,01868184 | 1,46196684 |
| ENSG00000211598 | IGKV4-1 | 0,0106297 | 1,46492155 |
| ENSG00000162512 | SDC3 | 0,00113328 | 1,46966711 |
| ENSG00000170476 | MZB1 | 0,00103592 | 1,47149892 |
| ENSG00000178445 | GLDC | 0,01308674 | 1,47423214 |
| ENSG00000117399 | CDC20 | 0,00212048 | 1,48208837 |
| ENSG00000173372 | C1QA | 0,01388837 | 1,50841027 |
| ENSG00000244437 | IGKV3-15 | 0,00122886 | 1,54663542 |
| ENSG00000183762 | KREMEN1 | 0,00737452 | 1,54721927 |
| ENSG00000243290 | IGKV1-12 | 0,04466114 | 1,5505125 |
| ENSG00000211592 | IGKC | 0,00674956 | 1,56344241 |
| ENSG00000142089 | IFITM3 | 0,04439731 | 1,593899 |
| ENSG00000243466 | IGKV1-5 | 0,00716235 | 1,60705193 |
| ENSG00000198019 | FCGR1B | 0,00077191 | 1,60717333 |
| ENSG00000198959 | TGM2 | 3,9847E-05 | 1,63929276 |
| ENSG00000010030 | ETV7 | 0,03397726 | 1,6441196 |
| ENSG00000211668 | IGLV2-11 | 0,01534385 | 1,65044264 |
| ENSG00000126709 | IFI6 | 0,01848499 | 1,66563259 |
| ENSG00000162772 | ATF3 | 0,00429156 | 1,68168762 |
| ENSG00000211666 | IGLV2-14 | 0,00748121 | 1,68420847 |
| ENSG00000002549 | LAP3 | 1,314E-05 | 1,68664886 |
| ENSG00000211625 | IGKV3D-20 | 0,010343 | 1,69967662 |
| ENSG00000214872 | SMTNL1 | 0,00092594 | 1,70153595 |
| ENSG00000239951 | IGKV3-20 | 0,00099468 | 1,70376736 |
| ENSG00000224373 | IGHV4-59 | 0,00066187 | 1,71642261 |
| ENSG00000241351 | IGKV3-11 | 0,00428901 | 1,73310568 |
| ENSG00000211673 | IGLV3-1 | 0,00260161 | 1,73545008 |
| ENSG00000211663 | IGLV3-19 | 0,00336806 | 1,74903012 |
| ENSG00000265531 | FCGR1CP | 0,0024052 | 1,75457526 |
| ENSG00000133106 | EPSTI1 | 0,02047234 | 1,77548155 |
| ENSG00000154451 | GBP5 | 0,00038159 | 1,77700903 |
| ENSG00000251546 | IGKV1D-39 | 0,00138069 | 1,80915277 |
| ENSG00000211967 | IGHV3-53 | 0,02009566 | 1,82055303 |
| ENSG00000211676 | IGLJ2 | 0,00212048 | 1,82526431 |
| ENSG00000211970 | IGHV4-61 | 0,02074849 | 1,82813746 |
| ENSG00000111331 | OAS3 | 0,02359755 | 1,82831518 |
| ENSG00000160932 | LY6E | 0,01641695 | 1,82842179 |
| ENSG00000136514 | RTP4 | 0,00060289 | 1,84178961 |
| ENSG00000150337 | FCGR1A | 0,00122886 | 1,84492028 |
| ENSG00000108387 | SEPT4 | 5,5166E-05 | 1,85462473 |
| ENSG00000188290 | HES4 | 0,00228916 | 1,89783126 |
| ENSG00000242076 | IGKV1-33 | 0,00077191 | 1,89884702 |
| ENSG00000239855 | IGKV1-6 | 0,02857777 | 1,90894923 |
| ENSG00000236056 | GAPDHP14 | 0,00190828 | 1,91142075 |
| ENSG00000159189 | C1QC | 0,01688748 | 1,91354267 |
| ENSG00000060656 | PTPRU | 0,04002964 | 1,94080624 |
| ENSG00000111291 | GPRC5D | 0,00671684 | 1,94972367 |
| ENSG00000211677 | IGLC2 | 0,00075013 | 1,95309917 |
| ENSG00000211648 | IGLV1-47 | 0,01557687 | 1,97202779 |
| ENSG00000175643 | RMI2 | 0,00174304 | 1,97214394 |
| ENSG00000137965 | IFI44 | 0,00633727 | 1,98693238 |
| ENSG00000173369 | C1QB | 0,01425641 | 2,0054614 |
| ENSG00000120217 | CD274 | 3,2429E-06 | 2,05196415 |
| ENSG00000211941 | IGHV3-11 | 0,00314473 | 2,06963169 |
| ENSG00000211644 | IGLV1-51 | 0,00038159 | 2,0722963 |
| ENSG00000211640 | IGLV6-57 | 0,00033618 | 2,0860453 |
| ENSG00000282639 | AC247036 | 0,02906322 | 2,09112771 |
| ENSG00000211670 | IGLV3-9 | 0,01425641 | 2,09912497 |
| ENSG00000211965 | IGHV3-49 | 0,00463956 | 2,12957238 |
| ENSG00000168062 | BATF2 | 0,00270829 | 2,14447387 |
| ENSG00000211653 | IGLV1-40 | 0,00028164 | 2,16051357 |
| ENSG00000149131 | SERPING1 | 0,00154988 | 2,16138131 |
| ENSG00000211905 | IGHJ1 | 0,00753807 | 2,16164035 |
| ENSG00000274576 | IGHV2-70 | 0,00119554 | 2,17671511 |
| ENSG00000134326 | CMPK2 | 0,00499214 | 2,1767217 |
| ENSG00000225573 | RPL35P5 | 0,0236241 | 2,18252245 |
| ENSG00000132170 | PPARG | 0,0023549 | 2,21895252 |
| ENSG00000211611 | IGKV6-21 | 0,0272186 | 2,26183123 |
| ENSG00000165949 | IFI27 | 0,00172766 | 2,28928653 |
| ENSG00000211662 | IGLV3-21 | 0,00699298 | 2,29377303 |
| ENSG00000197646 | PDCD1LG2 | 0,00141568 | 2,29646922 |
| ENSG00000187608 | ISG15 | 0,02880147 | 2,30743603 |
| ENSG00000173239 | LIPM | 0,00974904 | 2,31072636 |
| ENSG00000270550 | IGHV3-30 | 0,02074849 | 2,32437952 |
| ENSG00000211669 | IGLV3-10 | 0,0154983 | 2,33305438 |
| ENSG00000152766 | ANKRD22 | 3,2429E-06 | 2,38119028 |
| ENSG00000211664 | IGLV2-18 | 0,02275213 | 2,42998432 |
| ENSG00000115155 | OTOF | 0,001793 | 2,49222127 |
| ENSG00000211895 | IGHA1 | 0,0014133 | 2,55909929 |
| ENSG00000225492 | GBP1P1 | 0,00020341 | 2,75468728 |
| ENSG00000225523 | IGKV6D-21 | 0,00077191 | 3,14607258 |
| ENSG00000211655 | IGLV1-36 | 0,00050811 | 3,57602097 |
| ENSG00000253818 | IGLV1-41 | 0,00020352 | 4,87575438 |

*Genes are identified by their Gene symbol and the corresponding Ensembl ID. All genes included in the list presented significant differences (adjusted p value <0.01) between the groups being compared and an absolute Log2 fold change <1. Genes with Log2CF > 1 are up-regulated and Lof2FC < -1 down-regulated in TB-like. Differential expression was calculated using the R package DESeq2 with the default parameters (p-values were calculated by the Wald test and corrected for multiple testing using the Benjamini and Hochberg method).*
